# Controllable properties and versatile dynamics of meron topological magnetism in van der Waals multiferroic CuCrP_2_S_6_

**DOI:** 10.1016/j.isci.2025.113291

**Published:** 2025-08-05

**Authors:** Qirui Cui, Yuqing Ge, Xiaocheng Bai, Yasmine Sassa, Anna Delin

**Affiliations:** 1 Department of Applied Physics, School of Engineering Sciences, KTH Royal Institute of Technology, AlbaNova University Center, SE-10691 Stockholm, Sweden; 2 Swedish e-Science Research Center, KTH Royal Institute of Technology, 10044 Stockholm, Sweden; 3 School of Science, Xi’an University of Posts and Telecommunications, Xi’an 710121, China; 4 Wallenberg Initiative Materials Science for Sustainability (WISE), KTH Royal Institute of Technology, SE-10044 Stockholm, Sweden

**Keywords:** Physics, Condensed matter physics, Magnetism

## Abstract

The ability to efficiently control topological magnetism is crucial for advancing technological applications and deepening our understanding of magnetic systems. Although emerging van der Waals (vdW) multiferroics present a promising frontier for energy-efficient spin manipulation, the control of topological magnetism remains challenging due to its scarcity in multiferroics. Here, we demonstrate that highly tunable merons and antimerons emerge in monolayer multiferroic ${\text{CuCrP}}_{2}S_{6}$ (CCPS). The antiferroelectric-to-ferroelectric (AFE-FE) transition enhances exchange couplings, notably reducing meron density and increasing meron size during cooling. Merons exhibit unique dynamics, characterized by nontrivial attraction and annihilation processes, which generates distinct long-lived spin waves and reduces meron number difference between AFE and FE phases until they vanish. Importantly, ultrafast laser pulses can induce ferroelectricity-tunable merons from a uniform in-plane magnetization, re-leading to a large difference in meron density between the AFE and FE phases. These findings enhance our understanding of topological magnetism and open up exciting avenues for controlling the properties and dynamics of topological states through electrical and optical methods.

## Highlights

• The topological magnetic merons can emerge spontaneously in multiferroic CuCrP_2_S_6_
• The meron density and size are controlled by ferroelectricity
• The meron-antimeron interactions and consequent spin wave generation are analyzed
• The topological merons morphology is effectively controlled by ultrafast leaser excitation

## Graphical abstract

**Figure:**
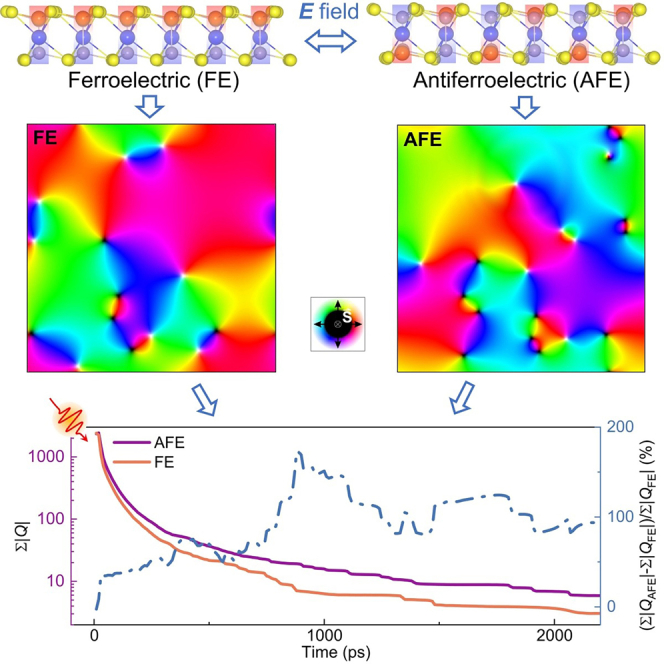

## Introduction

Topological spin configurations with intrinsic swirling spin structures, such as skyrmions and merons, are correlated with many interesting phenomena in condensed matter, including superconductivity, the topological Hall effect, multiferroicity, and more.^1^^,^^2^^,^^3^^,^^4^^,^^5^ The topological spin texture is associated with an energy barrier, resulting in enhanced robustness of these spin configurations against external perturbations. This inherent stability makes them ideally suited as carriers of information bits in nonvolatile high-density and energy-efficient memory devices.^6^^,^^7^^,^^8^^,^^9^ Toward technological applications, tremendous efforts have been devoted to manipulating topological spin configurations with high speed and low dissipation. One promising approach is the application of an electric field to various magnetic heterostructures, such as ferromagnetic metal/heavy metal, oxides, and ferroelectric layers.^10^^,^^11^^,^^12^^,^^13^^,^^14^^,^^15^ The electric field can modulate electronic states, the interfacial atomic distribution, or induce inverse magneto-mechanical effects, ultimately leading to tunability of the magnetic parameters and the topological magnetism. Another important approach is utilizing ultrafast laser pulses to control spin dynamics by rapidly heating electrons, inducing effective magnetic fields, driving coherent spin precession, etc.^16^^,^^17^^,^^18^^,^^19^^,^^20^^,^^21^ This process excites the electronic structure and enables the system to overcome energy barriers between different magnetic phases. Notably, ultrafast laser pulses have been applied to realize the creation, annihilation, switching, and spatial manipulation of different topological spin textures in magnetic thin films.^22^^,^^23^^,^^24^^,^^25^^,^^26^

The vdW magnets with long-range magnetic order even at monolayer provide an intriguing platform for exploring topological magnetism at atomic thickness.^27^ Specifically, a variety of spin configurations – including chiral domain walls, skyrmion, merons, and antimerons – have been recently observed in vdW systems like ${\text{CrCl}}_{3}$, ${\text{CrBr}}_{3}$, ${\text{CrGeTe}}_{3}$, ${\text{Fe}}_{3}{\text{GeTe}}_{2}$, and ${\text{Fe}}_{5}{\text{GeTe}}_{2}$.^28^^,^^29^^,^^30^^,^^31^^,^^32^^,^^33^^,^^34^^,^^35^^,^^36^^,^^37^ These topological spin configurations, however, are insensitive to the electric field, as the electric field cannot directly break time-reversal symmetry. The pursuit of electric field-controlled magnetism further highlights the multiferroics which combine ferroelectricity and magnetism as promising candidates. Despite the multiferroicity has recently been reported in vdW magnets ${\text{NiI}}_{2}$ and ${\text{CuCrSe}}_{2}$, achieving the ferroelectric control of magnetism remains elusive in these systems.^38^^,^^39^^,^^40^ Interestingly, ${\text{CuCrP}}_{2}S_{6}$ (CCPS) presents a unique opportunity for 2D ferroelectricity-magnetism interactions,^41^^,^^42^^,^^43^^,^^44^^,^^45^^,^^46^^,^^47^^,^^48^ as evidenced by the experimentally observed AFE-FE transitions,^49^^,^^50^ net magnetization in odd number of layers,^51^ and electrically tunable interlayer couplings.^52^ However, the potential for realizing topological magnetism in CCPS has been overlooked, due to the prevailing belief that it exhibits only collinear magnetism. Consequently, crucial aspects such as the ferroelectric tunability and spin dynamics associated with potential topological magnetism is unexplored. Additionally, while ultrafast lasers have been utilized to switch magnetization^53^ and control magnon dynamics^54^ or topological phases^55^^,^^56^^,^^57^ in vdW ferromagnets, the response of the magnetic order in vdW multiferroic systems to laser excitation still remains to be investigated.

In this work, we demonstrate the emergence of controllable topological merons and antimerons, and reveal their unique dynamics in monolayer CCPS. The AFE-FE transition of CCPS leads to a decrease in meron density and an increase in meron size during zero-field cooling, driven by enhanced exchange couplings. Merons and antimerons exhibit mutual attraction based on their topological charge. As a function of time, they therefore tend to draw closer to each other until they eventually annihilate. The pair annihilation further generates two distinct long-lived spin waves, one spiral and one unidirectional. During prolonged spin evolution, the pair annihilation tends to reduce the meron number difference between the AFE and FE phases, and leads to the disappearance of merons. Additionally, we demonstrate that the AFE-FE transition enhances the mutual attraction between the meron and the antimeron within a single pair, as confirmed by the continuum model. Finally, we show that ultrafast laser pulses can excite merons and antimerons in CCPS, even from a uniform in-plane magnetization state. The density and size of excited merons are also strongly correlated with the AFE and FE phases. The variations in spin configurations induced by the laser pulse are clearly reflected in the spin correlation. These findings thus provide novel approaches for realizing tunable topological magnetism and investigating ultrafast spin dynamics in realistic ultrathin systems.

## Results and discussion

### Structural and magnetic properties of CCPS

**Figure 1:**
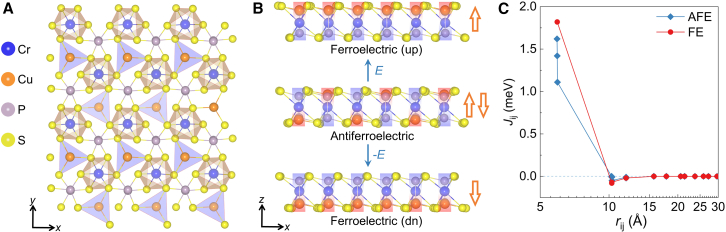
Crystal and magnetic properties of CCPS (A) Top and (B) side views of monolayer CCPS. The transition between the AFE and FE phases (B) is realized by shifting the positions of the Cu atoms. (C) Heisenberg exchange coupling parameters as a function of the Cr pair distance. The strength of the exchange couplings is enhanced due to the AFE-FE transition.

Bulk CCPS exhibits a centrosymmetric crystal structure with a space group of C2/c at room temperature, transitioning to a non-centrosymmetric phase with space group Pc below a temperature of 145 K.^48^ In contrast, a monolayer of CCPS has space group P1 (Figures 1A and 1B). Here, Cr atoms occupy positions within sulfur octahedra, forming a triangular lattice. The slight off-center Cu positions in the sulfur octahedra produces nearly flat ${\text{CuS}}_{3}$ triangles in a single layer. Alternating Cu atoms along the *x* axis generate antiparallel electric dipoles, characteristic of an AFE state. More specifically, the AFE CCPS indicates that the Cu ions alternately occupy the upper and lower positions, resulting in an ordered arrangement of localized opposite polarizations, and the absence of spontaneous macroscopic polarization. Under a perpendicular electric field, Cu atoms can be displaced to the same side, corresponding to the FE state.^41^^,^^50^ The calculated magnitude of the out-of-plane electric dipole of a single layer is around 0.21 eÅ per unit cell.

The magnetism in CCPS originates mainly from the Cr atoms, which have a magnetic moment of around 3.29 $\mu_{B}$/atom, as obtained from DFT calculations. The following spin Hamiltonian is adopted for further describing the magnetic properties: $H=-\sum_{\left\langle ij\right\rangle }J_{ij}S_{i}\cdot S_{j}-\sum_{i}K{\left(S_{i}^{z}\right)}^{2}-\sum_{\left\langle ij\right\rangle }D_{ij}\cdot \left(S_{i}\times S_{j}\right)$, where *J* is the Heisenberg exchange couplings, *K* is the magnetic anisotropy and D is the Dzyaloshinskii–Moriya interaction. We adopt the sign convention where J>0 favors ferromagnetic exchange couplings, and K<0 promotes in-plane magnetization. The values of the exchange coupling parameters in the spin Hamiltonian are obtained with the Liechtenstein-Katsnelson-Antropov-Gubanov (LKAG) formula, which is based on real-space Green’s functions (see STAR Methods).^58^^,^^59^^,^^60^^,^^61^^,^^62^ Figure 1C shows the dominant neighbor exchange couplings in the AFE phase, with $J_{1}=1.62$ meV, $J_{2}=1.42$ meV, and $J_{3}=1.12$ meV (blue dots). The distribution of these couplings among various Cr pairs is detailed in Figure S1. The antipolar structure with alternately arranged Cu atoms causes the Cr atoms to slightly shift from the center of the sulfur octahedra, rendering different exchange interactions.^63^ In contrast, the FE phase with a uniform polarization shows a single dominant exchange coupling parameter $J_{1}$ of 1.82 meV (see distribution in Figure S1) for all the six nearest neighbors (red dots). Clearly, the FE phase possesses an enhanced ferromagnetism compared to the AFE phase. Due to the large nearest neighbor Cr-Cr distance – of approximately 6 Å – the exchange interactions in CCPS are governed by superexchange couplings, such as Cr-S-S-Cr and Cr-S-Cu-S-Cr.^42^ In the FE phase, the electronic states are more localized due to the net electric polarization (Figures S2A and S2C). Moreover, the DOS peaks for S-3*p* and Cr-3*d* are distributed over nearly the same energy levels, and in the FE state, they are higher than in the AFE state. This indicates a stronger p−d hybridization, which leads to an enhanced superexchange coupling in the FE phase compared to the AFE phase.^64^^,^^65^^,^^66^ Moreover, similar exchange couplings with magnitude of 1–2 meV have been reported for the well-known chromium-based vdW magnet ${\text{CrI}}_{3}$^67^^,^^68^^,^^69^. By varying $U_{\text{eff}}$ from 1 to 4 eV, we demonstrate that our DFT computed exchange couplings in CCPS remain robust over this broad range, consistently yielding exchange couplings of 1–2 meV (see Table S1). Importantly, the key finding—enhanced ferromagnetism induced by the AFE-FE transition—persists for all tested $U_{\text{eff}}$ values. The magnetization melting, as modeled by Monte Carlo simulations, shows that the Curie temperature $T_{C}$ is increased from 17 to 22 K over the AFE-FE transition (Figures S2B and S2D). The magnetic anisotropy energies are K=−0.053 meV in the AFE phase and K=−0.045 meV in the FE phase, favoring an in-plane magnetization in both phases. Notably, the easy-plane magnetism has been experimentally observed in few-layer CCPS systems (≥ 1 layer) by nitrogen-vacancy center microscopy^51^ and magnetic tunneling measurements.^52^ Given the low crystal symmetry of CCPS, it is essential to evaluate the potential influence of the DMI that can favor the noncollinear spin configurations. Specifically, the breaking of mirror and in-plane rotation symmetries implies that the DMI can be decomposed into three components^4^^,^^70^^,^^71^: $D_{ij}=d_{⊥}\left(r_{ij}\times z\right)+d_{∥}r_{ij}+d_{z}z$. The DFT calculations using Green’s function methods^59^^,^^60^^,^^61^ indeed provide explicit magnitudes for all components.^72^ However, due to the absence of heavy elements with strong spin-orbit coupling, the DMI in CCPS is very weak and at least two orders of magnitude smaller than the Heisenberg exchange couplings.

### Meron and antimerons in CCPS

**Figure 2:**
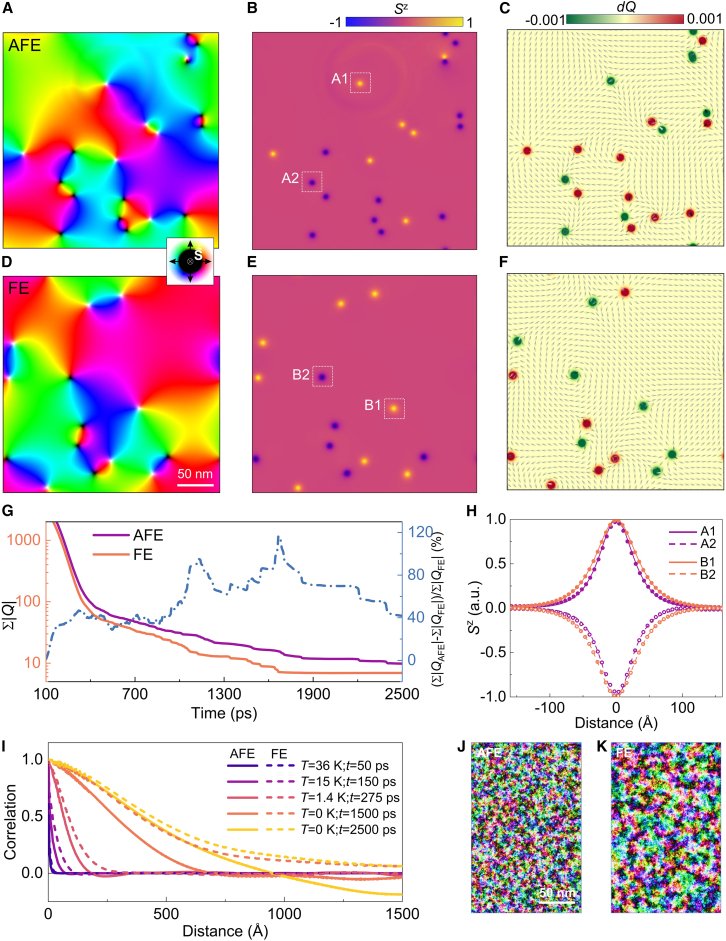
Topological spin configurations in CCPS with FE and AFE phases (A) In-plane and (B) out-of-plane spin components of the AFE phase at t=2500 ps and T=0 K. The simulation zone, with periodic boundary conditions, is set to 300 × 300 nm. (C) The corresponding topological charge density, clearly illustrating the solitonic nature of the observed merons and antimerons. (D–F) correspond to (A)-(C), but for the FE phase. (G) Summed-up absolute values of the topological charge ∑|Q| (solid lines) and relative magnitude difference between the AFE and FE phases (the dashed line) as functions of evolution time. (H) The $S^{z}$ components of merons and antimerons, highlighted by the white dashed squares in (B) and (E). The dots represent data obtained from atomic spin model simulations, while the lines correspond to fitting results using the formula: $S^{z}=\sin\left(\arctan\left(1/\sinh\left(r/w\right)\right)\right)$. The diameter is defined as the $S^{z}<0.1$ (I) Spin correlation functions at different stages of zero-field cooling. (J) and (K) Snapshots of spin configurations of the AFE and FE phases at T=15 K during zero-field cooling.

Based on the resolved spin Hamiltonian, the effective magnetic field experienced by each spin is defined as $H_{\text{eff}}^{i}=-\frac{1}{\mu_{s}}\frac{\partial H}{\partial S_{i}}$. The spin dynamics at various temperatures is then described by the Landau–Lifshitz–Gilbert (LLG) equation (see STAR Methods). To explore potential stable spin configurations, the spins are initiated in a random state at a high temperature $T\gg T_{c}$. The system is then gradually cooled down to 0 K within 400 ps, followed by continuous time-series evolutions. Interestingly, the magnetization does not fully lie in the plane but retains some out-of-plane components, which is observed in both the AFE and FE phases (see Video S1). As illustrated by the light and dark blobs in Figures 2A and 2B, merons and antimerons emerge randomly along the boundary of the in-plane domain, without any clear indication of a meron-antimeron lattice formation. These localized spin textures in two-dimensional systems possess the topological charge computed as $Q=\frac{1}{4\pi }\int S\cdot \left(\frac{\partial S}{\partial x}\times \frac{\partial S}{\partial y}\right)dxdy\text{.}$ The topological charge *Q* for merons in a continuous film takes half-integer values, which corresponds to half wrapping of the $S^{2}$ spin space over the 2D plane, typically from the pole to the equator. Figure 2C displays that each meron or antimeron possesses the localized topological charge, i.e., Q=±0.5(∓0.5) for meron (antimeron) with up and down core magnetization. The ratio of merons vs. antimerons is maintained at 1:1 conserving the total topological charge. Similar to the AFE phase, merons and antimerons also emerge in the FE phase (Figures 2D–2F) after the zero-field cooling. Interestingly, the FE phase exhibits a lower meron density compared to the AFE phase. For instance, the total number of merons and antimerons is $N_{\text{AFE}}$ = 20 and $N_{\text{FE}}$ = 14 at *t* = 2500 ps (Figures 2B and 2E), corresponding to the density of 222 $\mu m^{-2}$ and 156 $\mu m^{-2}$, respectively. We further introduce the descriptor ∑|Q| to quantify the time-dependent meron density variation since the size of simulation region is fixed. This quantification can represent the number of merons when the system is composed of meron solitons instead of randomize spin configuration. As illustrated in Figure 2G, the meron density in the FE phase is obviously less than that of the AFE phase during the spin evolution, can be over 100 % and remaining ∼ 40 % less through the simulated time window.

Video S1. The temporal evolution of spin configurations in the FE and AFE phases, spanning 0 to 2500 ps, as obtained from field-cooling simulations

The swirling spin textures indicate that merons are located at the boundaries between in-plane magnetic domains with opposite spin polarization (Figures 2A and 2D). The FE phase exhibits stronger exchange couplings than the AFE phase, resulting in a greater effective in-plane magnetization field (see snapshots in Figure S3). By counteracting thermal fluctuations (T≠0), this field promotes the formation of larger domains during zero-field cooling in the FE phase, in contrast to the smaller domains that can accommodate more merons in the AFE phase (Figures 2J and 2K). The analysis above explains the observed difference in meron numbers shown in Figure 2G.

Another notable feature is that merons in the FE phase exhibit a larger size compared to those in the AFE phase (Figure 2H), as zoomed by Figure S4. Specifically, the average meron diameters^73^ increases from ∼ 12.87 nm (± 0.49 nm) to ∼ 15.88 nm (± 0.68 nm) after the AFE-FE transition, representing a 52 % area enhancement. The standard deviations in the parenthesis are obtained from averaging over meron solitons in the same frame. This enlargement is also attributed to stronger exchange interactions reducing the spin canting angle between neighboring spins. A smaller canting angle extends the spatial extent of the chiral spin structure, leading to the meron size expansion. These results clearly demonstrate that the AFE-FE phase transition in CCPS effectively controls both the meron density and meron morphology during zero-field cooling. Moreover, the merons and antimerons persist for over several thousand ps (Figure 2G), which is much longer than those observed in ferromagnetic iron^24^ and nickel-iron alloys.^25^ This extended lifetime enhances the suitability for imaging ferroelectricity-tunable spin configurations by magneto-optical techniques, such as Lorentz transmission electron microscopy (LTEM)^24^^,^^25^ and magnetic transmission X-ray microscopy.^74^ We also recommend preparing high-quality samples with minimal substrate interactions to preserve the intrinsic magnetic properties of CCPS. Moreover, to accurately resolve topological textures in the 10–20 nm range, a spatial resolution better than 10 nm in the chosen microscopy technique is essential. We would like to note that the direct measurement of their time-resolved trajectories is essential, rather than stabilizing a lattice structure. Ultrafast laser LTEM, which provides spatial resolution down to 2 nm,^75^^,^^76^^,^^77^^,^^78^ is well-established for studying magnetic texture dynamics. Notably, Möller et al. captured the time-resolved dynamics and trajectories of meron solitons in polycrystalline permalloy at a temporal resolution of 500 ps, despite lacking both a stabilized meron lattice and DMI.^78^ Given that the merons predicted in CCPS exhibit lifetimes exceeding 20 ns (see later discussions) and have average diameters around 15 nm, LTEM is clearly suitable for exploring these topological solitons.

Figure 2I shows the spin correlation $C\left(r\right)=\left\langle S_{1}\left(r_{1}\right)\cdot S_{2}\left(r_{2}\right)\right\rangle /{\left|S\right|}^{2}$ during the zero field-cooling. At *T* = 35 K, *C* decays rapidly and exponentially to zero with increasing distance, indicating that the system is in the paramagnetic phase with no detectable spin order. In contrast, the decay of *C* becomes much slower as *T* decreases toward 0 K, indicating the emergence of quasi-long-range order due to the formation of merons and antimerons. Notably, *C* in the FE phases (dashed lines) remains higher than that in the AFE phases (solid lines), implying a more ordered spin configuration. This difference appears at the very beginning of the spin evolution, with larger in-plane domains observed in the FE phase (Figures 2J and 2K). We also find that *C* can become negative in the AFE phase, while it keeps positive in the FE phase (Figure 2I). This implies that compared to the FE phase, the AFE phase consists of smaller multidomains where neighboring domains exhibit opposite spin polarization.

A key question that arises is how these topological spin configurations form despite a very weak DMI.^72^ Notably, ferroelectricity-tunable merons also emerge even when the DMI is not included in the atomistic spin model (Figure S5). During cooling, short-range Heisenberg exchange interactions promote magnon localization, i.e., the nucleation and growth of small, non-equilibrium magnetic textures.^78^ This process leads to the formation of magnetic droplets with unstable spin configurations that may orient perpendicularly to the easy plane.^79^^,^^80^ These droplets split, merge, and decay, ultimately leading to the emergence of merons and antimerons as thermal disturbances dissipate (Video S1). The intrinsic topological protection of these spin textures, manifested as a finite potential barrier protecting against immediate decay, in turn renders them robust against external perturbations. In the above dynamic process, the finite exchange couplings play a crucial role in stabilizing the meron with out-of-plane core magnetization. As shown in Figure S3, it is evident that the exchange field along the *z*-direction aligns with and supports the spin component $S^{z}$. As a comparison, by artificially setting a zero exchange coupling constant while keeping all other magnetic parameters unchanged, the out-of-plane magnetization is suppressed dramatically (Figure S6) and no topological spin texture appears upon the same cooling procedure to 0 K, as shown in Video S2. The weak in-plane magnetic anisotropy is also essential for the formation of merons and antimerons. In contrast, when the system is in an out-of-plane magnetic anisotropy, only multiple domains separated by hybridized chiral domain walls are observed (Figure S7).

Video S2. The temporal evolution of spin configurations in the AFE phase, spanning 0 to 2500 ps, as obtained from field-cooling simulations The magnitudes of exchange couplings are artificially set to be zero.

Using atomistic spin model simulations, we tracked the time evolution of the $S^{z}$ component in a bilayer CCPS system in its ground antiferroelectric (AFE) phase (see Video S5). The dominant interlayer exchange, $J_{c}$ = -0.12 meV, obtained from DFT calculations, favors antiferromagnetic coupling. After zero-field cooling, antiferromagnetic merons emerge. Due to the cancellation of the Magnus force in the bilayer, the typical *Q*-dependent dynamics of monolayer systems is absent in the low-velocity regime. Moreover, the evolution of meron pairs does not exhibit chiral or unidirectional motion, which leads to a faster annihilation process; in contrast, in monolayer systems, annihilations of the meron pairs are slowed down by the non-zero Magnus force, which causes their trajectories to significantly deviate. Consequently, the annihilation process in the bilayer generates nearly circular spin wave excitations, rather than the spiral or unidirectional patterns typical in monolayers. Moreover, since the Magnus force scales with velocity, the antiferromagnetic spin textures in the top and bottom layers eventually decouple by the end of the annihilation process. Our results clearly demonstrate that, despite exhibiting different dynamics, the emergence of topological merons is not limited to monolayer systems but is also observed in multilayer configurations.

Video S5. The temporal evolution of spin configurations in the antiferromagnetic bilayer CCPS with AFE phases, spanning 0 to 2500 ps, as obtained from field-cooling simulations

### Emission of spiral and unidirectional spin wave

**Figure 3:**
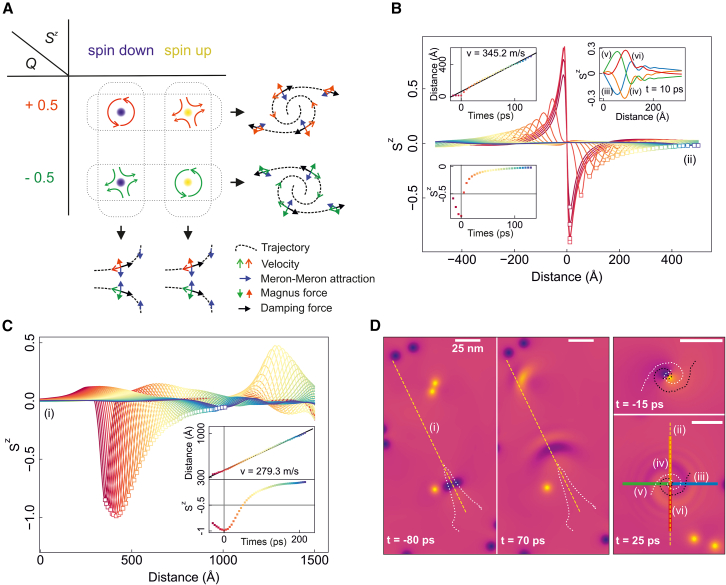
Topological charge-dependent meron dynamics and spin-wave emissions (A) Schematic representation of the *Q*-dependent binding processes between merons and antimerons. The dominant difference is that the pair with same *Q* experience a same-handed Magnus force, while the pair with opposite *Q* experience an opposite-handed Magnus force. The resultant $S^{z}$ components along cuts (i-vi) in (D) after the pair annihilation with (B) same and (C) opposite *Q*, indicating the spiral and unidirectional spin waves, respectively. The insets show the peak position and peak magnitude of $S^{z}$ as a function of evolution time. The propagated directions of $S^{z}$ shown in (B) and (C) are labeled by yellow dashed lines in (D), which shows selected real-space spin configurations. The spiral feature is further illustrated in the $S^{z}$ spatial distribution along four directions, as shown in the upper right panel of (B). The white and black dashed lines indicate the (anti)meron binding trajectory extracted by Trackpy.^83^

Next, we discuss the spin dynamics in CCPS. The binding between merons and antimerons reduces the system’s total energy, suggesting the presence of effective attractive interaction between meron and antimeron at finite range (see later discussions). Furthermore, topological quasiparticles experience Magnus forces proportional and perpendicular to their velocity. Based on the definition of *Q*, (anti)merons with positive charges experience a right-handed Magnus force with respect to their velocity, and vice versa for those with negative *Q*. As a result, the (anti)meron dynamics involve three competing forces: meron-antimeron attraction, Magnus force, and damping force antiparallel to the velocity. When approaching each other, (anti)merons accelerates and the Magnus force becomes significant as the velocity increases. This charge-dependent Magnus force thus leads to two scenarios: (1) merons and antimerons with the same *Q* approach each other along spiral trajectories since the Magnus forces deviate their trajectories away from the initial attractive direction and (2) merons and antimerons with opposite *Q* align in the same direction driven by their opposite Magnus forces, as elucidated in Figure 3A. Upon annihilation, different types of spin waves are emitted depending on the dynamics of the merons. Process (1) emits a spiral spin wave, where the $S^{z}$ components spread out with equal amplitudes but opposite signs in opposite directions (Figure 3B). In contrast, process (2) produces a unidirectional spin wave, where the wave front propagates exclusively in a single direction (Figure 3C). The spin configuration snapshots of these two spin waves are shown in Figure 3D. Based on the time-dependent $S^{z}$ peak intensity (Figures 3B and 3C), the spin wave lifetimes—defined as the time required for the peak intensity to decrease to 10% of its maximum—are estimated to be 50 ps for the spiral wave and 150 ps for the unidirectional wave. The corresponding spin wave velocities are 345.2 m/s and 279.3 m/s, respectively. This lifetime is comparable to that observed in vdW magnets ${\text{CrI}}_{3}$ and ${\text{MnBi}}_{2}{\text{Te}}_{4}$, i.e., on the order of picoseconds.^81^^,^^82^ This suggests the feasibility of experimentally measuring the spontaneous spin waves.

### Long-time spin configurations evolution

**Figure 4:**
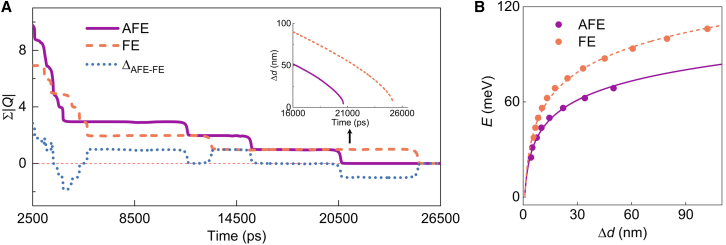
Long-time spin evolution (A) ∑|Q| as a function of time from 2500 to 26500 ps. The blue dotted line shows the difference of ∑|Q| between the AFE and FE phases. The inset illustrates the distance between the last meron and antimeron pair as a function of evolution time. (B) Total energy of the system as a function of pair distance. The zero point is defined as the system energy with a uniform in-plane magnetization, as determined by atomistic spin model simulations. The fitting lines are obtained from the continuum model.

The unique dynamics of merons and antimerons, including their attraction and annihilation, prompt further exploration of spin evolution over much longer time (refer to Video S3). Following pair annihilation, the meron number difference between the FE and AFE phases also tends to decrease, with the maximum difference reduced to a single meron-antimeron pair after ∼6 ns (Figure 4A). Notably, merons can persist for over 20 ns in both phases. Specifically, a single meron-antimeron pair remains in the FE and AFE systems after ∼13.2 ns and ∼15.5 ns, respectively. The total energy of the system thus depends on the interactions between the meron and antimeron. As they approach each other, the total energy exhibits a logarithmic-scale decay (Figure 4B), suggesting a correspondingly increasing attractive interaction. Due to the highly localized nature of the meron, this asymptotic interaction is primarily governed by regions far from their cores, where the exchange couplings among the in-plane swirling spins play a dominant role. The enhanced exchange couplings in the FE phase naturally result in a stronger attractive interaction between the meron and antimeron compared to that in the AFE phase (Figure 4B). Due to a negligibly small magnetic anisotropy, the continuum model which treats the spin configuration as a continuous field in space^84^^,^^85^ gives the energy expression of a meron-antimeron pair as^86^: $E=4\pi J\text{In}\left(d/a_{0}\right)$, where J is the effective exchange coupling strength and $a_{0}$ is the critical separation distance avoiding the divergence. We adopt the $J=\left(J_{1}+J_{2}+J_{3}\right)/3=1.39$ meV for the AFE phase, and $J=J_{1}=1.82$ meV for the FE phase. This continuum model shows good agreement with the atomistic spin model simulations (see solid and dashed lines in Figure 4B). Our findings therefore suggest that the AFE-FE phase transition can effectively modulate the meron-antimeron interactions in CCPS. Counterintuitively, the last remaining pair persists longer in the FE phase. This is because the historical larger pair distance (see inset of Figure 4A) is maintained after the evolution of the disordered multi-meron state, rendering a longer time for them to approach each other and annihilate. Therefore, the lifetime of the last pair of (anti)merons does not directly reflect the relative coupling strengths in the two phases. This also explains the fluctuations in the meron density relationship observed after 3000 ps (Figure 4A). The higher density in the AFE phase, retained from the cooling process, suggests a shorter averaged pair distance, which could facilitate their annihilation.

Video S3. The long-term evolution of spin configurations in the FE and AFE phases, spanning 2500 to 26500 ps, is simulated at 0 K

### Laser-excited merons and antimerons

**Figure 5:**
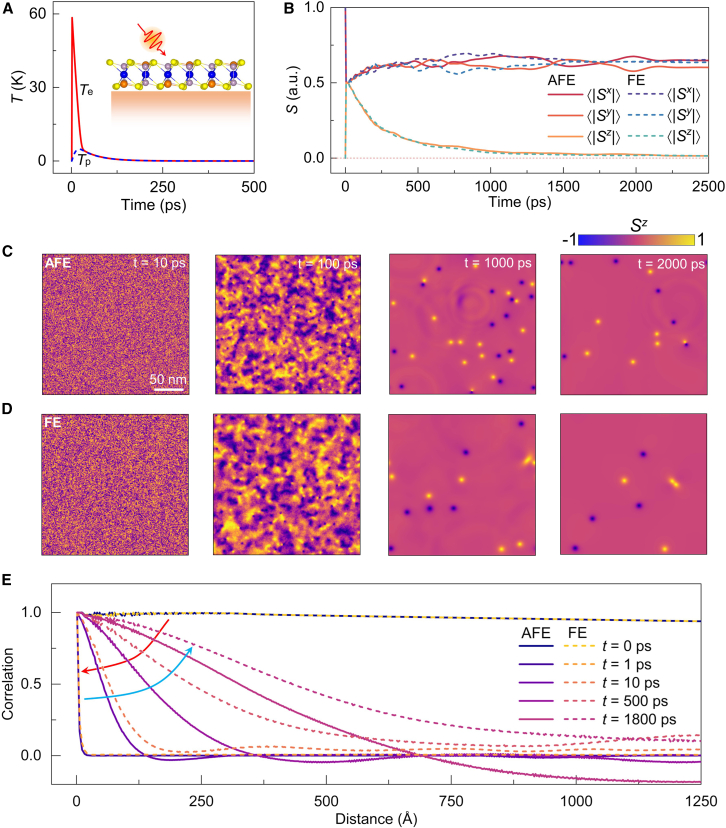
Stimulated ultrafast laser-pulse induced merons and antimerons (A) The electron and phonon temperature response to a 100 fs laser pulse. (B) Time evolution of the normalized averages of the absolute values of $S^{x}$, $S^{y}$, and $S^{z}$. (C and D) show the time-dependent spin configurations for the AFE and FE phases, respectively, in response to the laser. The simulation zone, with periodic boundary conditions, is set to 250 × 250 nm. (E) The spin correlation function induced by the laser pulse. The red arrow indicates the laser-induced rapid heating process from 0 fs to 100 fs, while the blue arrow marks the subsequent cooling process.

Being metastable states, merons and antimerons do not spontaneously emerge in CCPS from a uniform in-plane spin configuration. Despite the thermal fluctuations generating non-vanishing $S^{z}$ components, well-defined chiral spin configurations still do not develop in the time-series simulations at constant temperature, as shown in Figure S8. Interestingly, we find that an ultrafast laser pulse can efficiently excite merons in CCPS, and the resulting topological spin configurations are governed by the AFE and FE phases (see later discussions). The two-temperature model^87^ is employed to simulate the rapid thermal response of materials to a laser that induces heating (see STAR Methods). Under a laser pulse (100 fs) with a fluence of 0.01 mJ/${\text{cm}}^{2}$, the electron temperature ($T_{e}$) rapidly rises to approximately 58 K. Within a few picoseconds, $T_{e}$ equilibrates with the phonon temperature ($T_{p}$), after which the time evolution of both temperatures becomes indistinguishable (Figure 5A). Since the vdW layer is coupled to a heat sink, the deposited energy is efficiently dissipated via the substrate, leading to $T_{e}$ = $T_{p}$ ≈ 0.10 K after 220 ps. Figure 5B shows that the laser pulse rapidly quenches the magnetization at the onset of simulations. Importantly, during the cooling process, the $\left\langle \left|S^{z}\right|\right\rangle$ does not vanish immediately but gradually converges to a finite value. Laser-spin interactions are more clearly illustrated in Video S4. Following magnetization quenching, magnetic droplets nucleate rapidly and undergo splitting, merging, and diminishing processes within a few picoseconds. These processes result in the formation of more stable and well-defined merons as the system reaches thermal equilibrium (Figures 5C and 5D). The *Q*-dependent attraction and annihilation between merons and antimerons are accompanied by the emission of spin waves. Notably, the stronger exchange couplings in FE phase also lead to laser-induced merons with lower density and larger size. At t=2000 ps, the total meron number is 14 in the AFE phase and 8 in the FE phase, with diameters of ∼13.26 nm (± 0.58 nm) and ∼16.79 nm (± 0.58 nm), respectively. Identical results are observed when the laser power is increased to 0.04 mJ/ ${\text{cm}}^{2}$ (Figure S9). Despite the input of higher energy, after zero-field cooling, the merons in CCPS for both AFE and FE phases exhibit similar density and size to those observed under lower fluence. For example, at t=2000 ps, the total meron number is 16 in the AFE phase and 8 in the FE phase, with diameter of ∼13.11 nm (± 0.51 nm) and ∼16.97 nm (± 0.71 nm). Although pair annihilations reduce the meron number difference and eventually lead to a uniform in-plane magnetization, the laser pulse can reinduce a significant meron number difference between the AFE and FE phases (Figure S10). This indicates that periodic laser excitations can effectively sustain a higher meron density in the AFE phase compared to the FE phase.

Video S4. The temporal evolution of spin configurations following laser pulse excitation A two-temperature model is employed to describe the laser-induced magnetization quenching effects.

The tunability of the magnetic texture by laser pulses is also evident from the variation in spin correlation (Figure 5E). For the initial uniform state, the spin correlation function remains approximately constant. The spin correlation then sharply decreases to zero due to optical quenching. During zero-field cooling, the spin correlation undergoes an exponential decay, indicating the emergence of merons and antimerons. Additionally, it is important to assess whether the applied laser can trigger an AFE-FE phase transition. The minimum energy barrier for the transition, estimated by the CI-NEB method, is approximately 34 meV/f.u (∼ 394 K).^41^ The maximum laser-induced electron and phonon temperatures are 58 K and 5 K, respectively, which are well below this barrier.

Compared to zero-field cooling, meron formation remains evident even after laser excitation with a shorter (∼ 200 ps) cooling period. Extending the zero-field cooling time from 400 ps to 600 ps (Figure S11) does not suppress meron emergence, underscoring the robustness of our key results against cooling variations. As the system cools, exchange interactions induce out-of-plane magnetization within in-plane configurations, facilitating the nucleation of topologically nontrivial spin structures. Although longer experimental cooling (ns–ms) might enable complete thermal annealing, several factors stabilize merons under extended cooling. First, their intrinsic topological protection creates energy barriers that prevent annihilation, preserving them as metastable states. Second, experimental cooling is often nonuniform, with local temperature and rate variations promoting rapid quenching in specific regions and thereby enabling localized meron formation. We also find that increasing the fluence from 0.01 to 0.04 mJ/cm^2^ continues to yield FE-controlled topological merons, albeit requiring a longer cooling time (Figure S9). Similarly, when the fluence is fixed at 0.01 mJ/cm^2^, tuning the electron–lattice coupling factor *G* from $1.4\times 10^{15}$ to either $1.0\times 10^{15}$ or $1.8\times 10^{15}$ Wm^−3^ K^−1^ still results in the formation of topological merons in CCPS following laser-driven demagnetization (Figure S12). These results indicate that the laser-induced emergence of magnetic merons does not rely on finely tuned parameters, thereby reinforcing the physical realism of the two-temperature model simulations.

### Conclusions

In summary, we demonstrate that highly tunable merons and antimerons can emerge in the van der Waals multiferroic material CCPS. Despite the absence of prominent chiral interactions, such as DMI or spin frustration effects, nanoscale topological particles appear due to the competition between Heisenberg exchange interactions, in-plane magnetic anisotropy, and thermal fluctuations. The AFE-FE phase transition enhances the Heisenberg exchange couplings, which leads to a suppression in meron density and the enlarged meron size during the zero-field cooling. More specifically, the stronger exchange in the FE phase enhances the effective in-plane magnetization field, which can suppress thermal fluctuations and favors larger domains. In contrast, the weaker exchange in the AFE phase produces smaller domains that can host more merons, resulting in a higher meron density after the field-cooling. The larger size of merons in the FE phase also arises from stronger exchange interactions that reduce spin-canting angles and broaden the chiral spin structures. Unlike completely stochastic spin fluctuations, the dynamics of merons and antimerons are driven by the attraction force and *Q*-dependent Magnus force. The meron and antimeron pair with same *Q* spiral toward each other, while the pair with the opposite *Q* align in parallel when approaching. Interestingly, subsequent annihilation produces two distinct spin waves, spiral and unidirectional, with long lifetimes. Over long-time spin evolution, the annihilation decreases the meron number difference between the AFE and FE phases, ultimately resulting in the disappearance of merons (>20 ns). The reduced merons and the long lifetime of last remaining meron pair, in turn, make the system an ideal platform for investigating meron-antimeron interactions. The asymptotic attraction is accurately described by a continuum model and is notably enhanced by the AFE-FE phase transition. Finally, we show that merons and antimerons are excited by ultrafast laser pulses even when the CCPS has a uniform in-plane magnetization. The density and size of these topological particles depend on the AFE and FE phases, indicating that laser-spin coupling is modulated by the AFE-FE phase transition. The higher meron density in the AFE phase compared to the FE phase thus can be artificially achieved via laser excitations. The laser-induced magnetic phase transitions, from uniform magnetization to random magnetization and subsequently to the formation of merons and antimerons, is directly reflected in the spin correlation variation. Our results provide novel insights into the electrical and optical tunability of topological spin configurations and dynamics, and highlight the potential to apply vdW multiferroics for investigating topological magnetism toward spintronic or magnonic applications.

### Limitations of the study

We employ atomistic spin-model simulations with magnetic parameters derived from rigorous first-principles calculations to describe spin configurations in CuCrP_2_S_6_. Although these parameters are considered uniform and temperature-independent in our model, real materials may exhibit additional complexity due to environmental variations. Ultrafast laser excitation is modeled via a simplified yet widely accepted two-temperature model, primarily focusing on electron-lattice energy exchange. We assume that spin dynamics predominantly result from rapid thermal processes, and use fixed electron–lattice coupling constants and heat capacities as typical approximations, although potential direct spin-laser interactions are not explicitly considered.

Furthermore, the classical Landau–Lifshitz–Gilbert (LLG) equation employed here is a standard approach widely utilized in magnetic simulations, yet it inherently neglects quantum fluctuations and the discrete (quantized) nature of spins—effects which could become significant in low-dimensional systems and may influence phase transitions and topological stability. Our simulations focus specifically on single- and bilayer systems, which are highly relevant for two-dimensional materials research. However, for thicker or bulk samples, additional interlayer coupling and magnetic dipolar interactions could potentially stabilize vertically extended topological structures or foster novel interlayer topological correlations. Hence, while our results are robust within the scope of idealized two-dimensional classical simulations, careful consideration should be given when extrapolating these findings to quantum or multilayer scenarios.

## Resource availability

### Lead contact

Further information and requests for any resources should be directed to and will be fulfilled by the Lead Contact Qirui Cui (qiruic@kth.se).

### Materials availability

This study did not generate new unique materials.

### Data and code availability

Data reported in this article are available from the lead contact upon reasonable request.

This article does not report any original code.

Any additional information required to reanalyze the data reported in this article or reproduce the results is available from the lead contact upon reasonable request.

## Acknowledgments

We thank Mathias Augustin, Qilong Cui, Alexander Edström, and Linghan Zhu for helpful discussions. Q.C. and A.D. are supported by the 10.13039/501100004359Vetenskapsrådet (grant numbers VR 2016-05980 and VR 2019-05304), and the 10.13039/501100004063Knut and Alice Wallenberg Foundation (grant numbers 2018.0060, 2021.0246, and 2022.0108). Y.G. is supported by the Swedish Foundation for Strategic Research (SSF) through the Swedish Neutron Graduate School (SwedNess) through the grant GSn15-0008. Y.S. is supported by the Knut and Alice Wallenberg foundation (grant numbers 2021.0150) and the 10.13039/501100004359Swedish Research Council through the Starting Grant DNR 2017-05078. This work was partially supported by the Wallenberg Initiative Materials Science for Sustainability (WISE) funded by the Knut and Alice Wallenberg Foundation. Some of the computations were enabled by resources provided by the National Academic Infrastructure for Supercomputing in Sweden (NAISS) and the Swedish National Infrastructure for Computing (SNIC) at NSC and PDC, partially funded by the Swedish Research Council through grant agreements no. 2022-06725 and no. 2018-05973.

## Author contributions

Q.C., supervision, conceptualization, methodology, data and results analysis, writing – original draft, review, and visualization; Y.G., methodology, data and results analysis, writing – original draft, and review; X.B., data and results analysis, and review; Y.S., data and results analysis, and review; A.D., supervision, writing – original draft, review, and visualization.

## Declaration of interests

The authors declare that this research is conducted without any commercial or financial relationships that can be perceived as potential conflicts of interest.

## STAR★Methods

### Key resources table

**Table:** 

REAGENT or RESOURCE	SOURCE	IDENTIFIER
**Software and algorithms**		

VASP	VASP Software GmbH	https://www.vasp.at
QuantumATK	Synopsys TCAD	https://www.synopsys.com/manufacturing/quantumatk.html
Vampire	Richard Evans Group from University of York	https://vampire.york.ac.uk

### Method details

#### First-principles calculations

The first-principles calculations are conducted using the Vienna Ab-initio Simulation Package (VASP) based on the density functional theory (DFT).^88^^,^^89^^,^^90^ The projector augmented wave (PAW) method is employed with recommended pseudopotentials.^91^ The cut-off energy of the plane-wave expansion is set to 400 eV. The exchange-correlation interactions are treated via the generalized gradient approximation (GGA), utilizing the Perdew-Burke-Ernzerhof (PBE) functional.^92^^,^^93^ A mesh of 12×6×1 Γ-centered k-points is dense enough for the Brillouin zone sampling of ${\text{CuCrP}}_{2}S_{6}$, and a vacuum space of 15 Å is adopted in the z-direction of the slab geometry to minimize inter-layer interactions. To describe the strong correlation effects of 3*d*ays electrons, the Hubbard *U* correction method is applied, with an $U_{\text{eff}}$ parameter set to 3 eV $U_{\text{eff}}$ values of 2 and 4 eV were also tested, and the key findings of this work were found to be robust against variations in $U_{\text{eff}}$. The crystal structures are fully relaxed until the Hellmann-Feynman forces on each atom were below $10^{-3}$ eV/Å, and the energy convergence criterion is set to $10^{-7}$ eV.

The Heisenberg exchange couplings are computed using the Liechtenstein-Katsnelson-Antropov-Gubanov (LKAG) formula, which is derived from real-space Green’s functions. The LKAG expression for Heisenberg exchange couplings reads: $J_{ij}=-\frac{1}{4\pi }\int_{-\infty }^{E_{f}}\text{ImTr}\left[\Delta_{ii}G_{ij}\left(\varepsilon ,R\right)\Delta_{jj}G_{ij}\left(\varepsilon ,-R\right)\right]d\varepsilon$, where R is the lattice vector connecting spin sites and Δ is the difference between the spin-up and spin-down parts of the Hamiltonian matrix. The DMI is an antisymmetric exchange interaction and is known as an important driver of topological magnetism in many systems. DMI arises due to spin-orbit coupling and such effects must therefore be considered when computing DMI exchange parameters. The real-space Green’s functions method is applied to extract the DMI strengths between neighboring spin pairs: $D_{ij}^{\mu }=2\text{Re}\left(A_{ij}^{0\mu }-A_{ij}^{\mu 0}\right)\frac{\Delta_{ii}\Delta_{jj}}{4}$, where $A_{ij}^{\mu \nu }=-\frac{1}{4\pi }\int_{-\infty }^{E_{f}}{\text{Tr}}_{L}\left[G_{ij}^{\mu }G_{ji}^{\nu }\right]d\varepsilon$. The QuantumATK (QATK) package, utilizing an expansion of electronic states through a linear combination of atomic orbitals (LCAO), is employed to obtain the Heisenberg exchange couplings and DMI.^94^ Additionally, the spin polarized GGA+*U* functional with $U_{\text{eff}}$ = 3 eV on the Cr 3*d* orbitals is applied in the QATK calculations. The magnetic anisotropy is calculated by comparing the self-consistent energy of the system with magnetization along the x and z directions.

#### Atomistic spin model simulations

Based on the DFT-resolved spin Hamiltonian, we perform atomistic spin model simulations by solving the LLG equation on each spin site: $\frac{\partial S_{i}}{\partial t}=-\frac{\gamma }{\left(1+\lambda^{2}\right)}\left[S_{i}\times H_{\text{eff}}^{i}+\lambda S_{i}\times \left(S_{i}\times H_{\text{eff}}^{i}\right)\right]$, where the gyromagnetic ratio $\gamma =1.76\times 10^{11}$ $T^{-1}s^{-1}$ and damping constant λ=0.1.^95^ In addition to Heisenberg exchange couplings and magnetic anisotropy, the DMI is incorporated in the atomistic spin model simulations. For the FE phase, we use the DMI vectors corresponding to the down-polarization state as a representation. It is important to note that the DMI has minimal influence on the resulting topological magnetism (Figure S5), as its magnitude is at least two orders smaller than the Heisenberg exchange couplings.^72^ For describing the ultrafast laser heating, we adopt the two-temperature model^87^: $C_{e}T_{e}\frac{\partial T_{e}}{\partial t}=-G\left(T_{e}-T_{l}\right)+S\left(t\right)$ and $C_{l}\frac{\partial T_{l}}{\partial t}=-G\left(T_{l}-T_{e}\right)-\kappa \nabla T_{l}$, where $C_{e}$ and $C_{l}$ is the electron and lattice heat capacity, and *G* is the electron-lattice coupling factor. S(t) is the time-dependent laser pulse power, and *κ* is the heat diffusion coefficient. The electronic temperature is then included in the LLG equation via the thermal field $B_{th}=\Gamma \left(t\right)\sqrt{2\lambda k_{B}T/\gamma \mu_{s}\Delta t}$. $C_{e}=550\;{\text{Jm}}^{-3}K^{-2}$, $C_{l}=1.5\times 10^{5}\;{\text{Jm}}^{-3}K^{-1}$ and $G=1.4\times 10^{15}\;{\text{Wm}}^{-3}K^{-1}$ estimated following the recent spin-phonon oscillation measurements in vdW magnets.^96^ The duration time of the laser pulse is set to 100 fs, and the heat-sink coupling constant is set to 50 ps. Monte Carlo simulations employing the Metropolis algorithm are performed to investigate the temperature dependence of the magnetization. For each temperature point, $8\times 10^{4}$ steps are conducted to ensure that the system reaches thermal equilibrium.

### Quantification and statistical analysis

Our study does not include statistical analysis or quantification.

## References

[1] Tokura Y., Kanazawa N. (2021). Magnetic skyrmion materials. Chem. Rev..

[2] Göbel B., Mertig I., Tretiakov O.A. (2021). Beyond skyrmions: Review and perspectives of alternative magnetic quasiparticles. Phys. Rep..

[3] Reichhardt C., Reichhardt C.J.O., Milošević M.V. (2022). Statics and dynamics of skyrmions interacting with disorder and nanostructures. Rev. Mod. Phys..

[4] Yang H., Liang J., Cui Q. (2022). First-principles calculations for Dzyaloshinskii–Moriya interaction. Nat. Rev. Phys..

[5] Everschor-Sitte K., Majumdar A., Wolk K., Meier D. (2024). Topological magnetic and ferroelectric systems for reservoir computing. Nat. Rev. Phys..

[6] Song K.M., Jeong J.S., Pan B., Zhang X., Xia J., Cha S., Park T.-E., Kim K., Finizio S., Raabe J. (2020). Skyrmion-based artificial synapses for neuromorphic computing. Nat. Electron..

[7] Amin O.J., Poole S.F., Reimers S., Barton L.X., Dal Din A., Maccherozzi F., Dhesi S.S., Novák V., Krizek F., Chauhan J.S. (2023). Antiferromagnetic half-skyrmions electrically generated and controlled at room temperature. Nat. Nanotechnol..

[8] Yokouchi T., Sugimoto S., Rana B., Seki S., Ogawa N., Shiomi Y., Kasai S., Otani Y. (2022). Pattern recognition with neuromorphic computing using magnetic field-induced dynamics of skyrmions. Sci. Adv..

[9] Xia J., Zhang X., Liu X., Zhou Y., Ezawa M. (2022). Qubits based on merons in magnetic nanodisks. Commun. Mater..

[10] Hsu P.-J., Kubetzka A., Finco A., Romming N., von Bergmann K., Wiesendanger R. (2017). Electric-field-driven switching of individual magnetic skyrmions. Nat. Nanotechnol..

[11] Ma C., Zhang X., Xia J., Ezawa M., Jiang W., Ono T., Piramanayagam S.N., Morisako A., Zhou Y., Liu X. (2019). Electric field-induced creation and directional motion of domain walls and skyrmion bubbles. Nano Lett..

[12] Dai B., Wu D., Razavi S.A., Xu S., He H., Shu Q., Jackson M., Mahfouzi F., Huang H., Pan Q. (2023). Electric field manipulation of spin chirality and skyrmion dynamic. Sci. Adv..

[13] Fillion C.-E., Fischer J., Kumar R., Fassatoui A., Pizzini S., Ranno L., Ourdani D., Belmeguenai M., Roussigné Y., Chérif S.-M. (2022). Gate-controlled skyrmion and domain wall chirality. Nat. Commun..

[14] Wang Y., Wang L., Xia J., Lai Z., Tian G., Zhang X., Hou Z., Gao X., Mi W., Feng C. (2020). Electric-field-driven non-volatile multi-state switching of individual skyrmions in a multiferroic heterostructure. Nat. Commun..

[15] Ba Y., Zhuang S., Zhang Y., Wang Y., Gao Y., Zhou H., Chen M., Sun W., Liu Q., Chai G. (2021). Electric-field control of skyrmions in multiferroic heterostructure via magnetoelectric coupling. Nat. Commun..

[16] Beaurepaire E., Merle J., Daunois A., Bigot J. (1996). Ultrafast spin dynamics in ferromagnetic nickel. Phys. Rev. Lett..

[17] Bergeard N., Hehn M., Mangin S., Lengaigne G., Montaigne F., Lalieu M.L.M., Koopmans B., Malinowski G. (2016). Hot-Electron-Induced Ultrafast Demagnetization in Co/Pt Multilayers. Phys. Rev. Lett..

[18] Mentink J.H., Hellsvik J., Afanasiev D.V., Ivanov B.A., Kirilyuk A., Kimel A.V., Eriksson O., Katsnelson M.I., Rasing T. (2012). Ultrafast spin dynamics in multisublattice magnets. Phys. Rev. Lett..

[19] Radu I., Vahaplar K., Stamm C., Kachel T., Pontius N., Dürr H.A., Ostler T.A., Barker J., Evans R.F.L., Chantrell R.W. (2011). Transient ferromagnetic-like state mediating ultrafast reversal of antiferromagnetically coupled spins. Nature.

[20] Razdolski I., Alekhin A., Ilin N., Meyburg J.P., Roddatis V., Diesing D., Bovensiepen U., Melnikov A. (2017). Nanoscale interface confinement of ultrafast spin transfer torque driving non-uniform spin dynamics. Nat. Commun..

[21] Ryan S.A., Johnsen P.C., Elhanoty M.F., Grafov A., Li N., Delin A., Markou A., Lesne E., Felser C., Eriksson O. (2023). Optically controlling the competition between spin flips and intersite spin transfer in a Heusler half-metal on sub–100-fs time scales. Sci. Adv..

[22] Je S.-G., Vallobra P., Srivastava T., Rojas-Sánchez J.-C., Pham T.H., Hehn M., Malinowski G., Baraduc C., Auffret S., Gaudin G. (2018). Creation of Magnetic Skyrmion Bubble Lattices by Ultrafast Laser in Ultrathin Films. Nano Lett..

[23] Zhang W., Huang T.X., Hehn M., Malinowski G., Verges M., Hohlfeld J., Remy Q., Lacour D., Wang X.R., Zhao G.P. (2023). Optical Creation of Skyrmions by Spin Reorientation Transition in Ferrimagnetic CoHo Alloys. ACS Appl. Mater. Inter..

[24] Eggebrecht T., Möller M., Gatzmann J.G., Rubiano da Silva N., Feist A., Martens U., Ulrichs H., Münzenberg M., Ropers C., Schäfer S. (2017). Light-Induced Metastable Magnetic Texture Uncovered by in situ Lorentz Microscopy. Phys. Rev. Lett..

[25] Fu X., Pollard S.D., Chen B., Yoo B.K., Yang H., Zhu Y. (2018). Optical manipulation of magnetic vortices visualized in situ by Lorentz electron microscopy. Sci. Adv..

[26] Tengdin P., Truc B., Sapozhnik A., Kong L., del Ser N., Gargiulo S., Madan I., Schönenberger T., Baral P.R., Che P. (2022). Imaging the Ultrafast Coherent Control of a Skyrmion Crystal. Phys. Rev. X.

[27] Gong C., Zhang X. (2019). Two-dimensional magnetic crystals and emergent heterostructure devices. Science.

[28] Lu X., Fei R., Zhu L., Yang L. (2020). Meron-like topological spin defects in monolayer CrCl_3_. Nat. Commun..

[29] Augustin M., Jenkins S., Evans R.F.L., Novoselov K.S., Santos E.J.G. (2021). Properties and dynamics of meron topological spin textures in the two-dimensional magnet CrCl_3_. Nat. Commun..

[30] Grebenchuk S., McKeever C., Grzeszczyk M., Chen Z., Šiškins M., McCray A.R.C., Li Y., Petford-Long A.K., Phatak C.M., Ruihuan D. (2024). Topological Spin Textures in an Insulating van der Waals Ferromagnet. Adv. Mater..

[31] Han M.-G., Garlow J.A., Liu Y., Zhang H., Li J., DiMarzio D., Knight M.W., Petrovic C., Jariwala D., Zhu Y. (2019). Topological Magnetic-Spin Textures in Two-Dimensional van der Waals Cr_2_Ge_2_Te_6_. Nano Lett..

[32] Wu Y., Francisco B., Chen Z., Wang W., Zhang Y., Wan C., Han X., Chi H., Hou Y., Lodesani A. (2022). A Van der Waals Interface Hosting Two Groups of Magnetic Skyrmions. Adv. Mater..

[33] Yang M., Li Q., Chopdekar R.V., Dhall R., Turner J., Carlström J.D., Ophus C., Klewe C., Shafer P., N’Diaye A.T. (2020). Creation of skyrmions in van der Waals ferromagnet Fe_3_GeTe_2_on (Co/Pd)_*n*_ superlattice. Sci. Adv..

[34] Birch M.T., Powalla L., Wintz S., Hovorka O., Litzius K., Loudon J.C., Turnbull L.A., Nehruji V., Son K., Bubeck C. (2022). History-dependent domain and skyrmion formation in 2D van der Waals magnet Fe_3_GeTe_2_. Nat. Commun..

[35] Zhang H., Raftrey D., Chan Y.-T., Shao Y.-T., Chen R., Chen X., Huang X., Reichanadter J.T., Dong K., Susarla S. (2022). Room-temperature skyrmion lattice in a layered magnet (Fe_0.5_Co_0.5_)_5_ GeTe_2_. Sci. Adv..

[36] Schmitt M., Denneulin T., Kovács A., Saunderson T.G., Rüßmann P., Shahee A., Scholz T., Tavabi A.H., Gradhand M., Mavropoulos P. (2022). Skyrmionic spin structures in layered Fe_5_GeTe_2_up to room temperature. Commun. Phys..

[37] Gao Y., Yin Q., Wang Q., Li Z., Cai J., Zhao T., Lei H., Wang S., Zhang Y., Shen B. (2020). Spontaneous (Anti)meron Chains in the Domain Walls of van der Waals Ferromagnetic Fe_5-x_GeTe_2_. Adv. Mater..

[38] Song Q., Occhialini C.A., Ergeçen E., Ilyas B., Amoroso D., Barone P., Kapeghian J., Watanabe K., Taniguchi T., Botana A.S. (2022). Evidence for a single-layer van der Waals multiferroic. Nature.

[39] Jiang Y., Wu Y., Zhang J., Wei J., Peng B., Qiu C.-W. (2023). Dilemma in optical identification of single-layer multiferroics. Nature.

[40] Sun Z., Su Y., Zhi A., Gao Z., Han X., Wu K., Bao L., Huang Y., Shi Y., Bai X. (2024). Evidence for multiferroicity in single-layer CuCrSe_2_. Nat. Commun..

[41] Qi J., Wang H., Chen X., Qian X. (2018). Two-dimensional multiferroic semiconductors with coexisting ferroelectricity and ferromagnetism. Appl. Phys. Lett..

[42] Colombet P., Leblanc A., Danot M., Rouxel J. (1982). Structural aspects and magnetic properties of the lamellar compound Cu 0.5Cr 0.5PS 3. J. Solid State Chem..

[43] Maisonneuve V., Cajipe V.B., Payen C. (1993). Low-temperature neutron powder diffraction study of CuCrP 2S 6: Observation of an ordered, antipolar copper sublattice. Chem. Mater..

[44] Maisonneuve V., Payen C., Cajipe V.B. (1995). On CuCrP 2S 6: Copper disorder, stacking distortions, and magnetic ordering. J. Solid State Chem..

[45] Lai Y., Song Z., Wan Y., Xue M., Wang C., Ye Y., Dai L., Zhang Z., Yang W., Du H., Yang J. (2019). Two-dimensional ferromagnetism and driven ferroelectricity in van der Waals CuCrP 2S 6. Nanoscale.

[46] Susner M.A., Rao R., Pelton A.T., Mcleod M.V., Maruyama B. (2020). Temperature-dependent Raman scattering and x-ray diffraction study of phase transitions in layered multiferroic CuCrP 2S 6. Phys. Rev. Mater..

[47] Io W.F., Pang S.-Y., Wong L.W., Zhao Y., Ding R., Mao J., Zhao Y., Guo F., Yuan S., Zhao J. (2023). Direct observation of intrinsic room temperature ferroelectricity in 2D layered CuCrP 2S 6. Nat. Commun..

[48] Aoki S., Dong Y., Wang Z., Huang X.S.W., Itahashi Y.M., Ogawa N., Ideue T., Iwasa Y. (2024). Giant modulation of the second harmonic generation by magnetoelectricity in two-dimensional multiferroic CuCrP 2S 6. Adv. Mater..

[49] Cho K., Lee S., Kalaivanan R., Sankar R., Choi K.-Y., Park S. (2022). Tunable ferroelectricity in van der Waals layered antiferroelectric CuCrP 2S 6. Adv. Funct. Mater..

[50] Ma Y., Yan Y., Luo L., Pazos S., Zhang C., Lv X., Chen M., Liu C., Wang Y., Chen A. (2023). High-performance van der Waals antiferroelectric CuCrP 2S 6-based memristors. Nat. Commun..

[51] Healey A.J., Rahman S., Scholten S.C., Robertson I.O., Abrahams G.J., Dontschuk N., Liu B., Hollenberg L.C.L., Lu Y., Tetienne J.-P. (2022). Varied magnetic phases in a van der Waals easy-plane antiferromagnet revealed by nitrogen-vacancy center microscopy. ACS Nano.

[52] Hu Q., Huang Y., Wang Y., Ding S., Zhang M., Hua C., Li L., Xu X., Yang J., Yuan S. (2024). Ferrielectricity controlled widely-tunable magnetoelectric coupling in van der Waals multiferroics. Nat. Commun..

[53] Dabrowski M., Guo S., Strungaru M., Keatley P.S., Withers F., Santos E.J.G., Hicken R.J. (2022). All-optical control of spin in a 2D van der Waals magnet. Nat. Commun..

[54] Gong Y., Yang Z., Teklu A., Xie T., Kern N., May A.F., McGuire M., Brennan C., Guo E.-J., Kuthirummal N. (2024). Efficient optical control of magnon dynamics in van der Waals ferromagnets. Ultrafast Sci..

[55] Strungaru M., Augustin M., Santos E.J.G. (2022). Ultrafast laser-driven topological spin textures on a 2D magnet. NPJ Comput. Mater..

[56] Kazemi M., Kudlis A., Bessarab P.F., Shelykh I.A. (2024). All-optical control of skyrmion configuration in CrI_3_monolayer. Sci. Rep..

[57] Khela M., Dabrowski M., Khan S., Keatley P.S., Verzhbitskiy I., Eda G., Hicken R.J., Kurebayashi H., Santos E.J.G. (2023). Laser-induced topological spin switching in a 2D van der Waals magnet. Nat. Commun..

[58] Liechtenstein A.I., Katsnelson M.I., Antropov V.P., Gubanov V.A. (1987). Local spin density functional approach to the theory of exchange interactions in ferromagnetic metals and alloys. J. Magn. Magn Mater..

[59] Szilva A., Costa M., Bergman A., Szunyogh L., Nordström L., Eriksson O. (2013). Interatomic exchange interactions for finite-temperature magnetism and nonequilibrium spin dynamics. Phys. Rev. Lett..

[60] Li D., Haldar S., Drevelow T., Heinze S. (2023). Tuning the magnetic interactions in van der Waals Fe_3_GeTe_2_heterostructures: A comparative study of ab initio methods. Phys. Rev. B.

[61] Szilva A., Kvashnin Y., Stepanov E.A., Nordström L., Eriksson O., Lichtenstein A.I., Katsnelson M.I. (2023). Quantitative theory of magnetic interactions in solids. Rev. Mod. Phys..

[62] Cui Q., Bai X., Delin A. (2024). Anisotropic magnon transport in van der Waals ferromagnetic insulators. Adv. Funct. Mater..

[63] Park C.B., Shahee A., Kim K.-T., Patil D.R., Guda S.A., Ter-Oganessian N., Kim K.H. (2022). Observation of spin-induced ferroelectricity in a layered van der Waals antiferromagnet CuCrP 2S 6. Adv. Electron. Mater..

[64] Anderson P.W. (1950). Antiferromagnetism. Theory of superexchange interaction. Phys. Rev..

[65] Goodenough J.B. (1955). Theory of the role of covalence in the perovskite-type manganites [La, M(II)]MnO_3_. Phys. Rev..

[66] Ashcroft N.W., Mermin N.D. (1976). Solid State Physics. Chapter 33.

[67] Pizzochero M., Yadav R., Yazyev O.V. (2020). Magnetic exchange interactions in monolayer CrI_3_ from many-body wavefunction calculations. 2D Mater..

[68] Sadhukhan B., Bergman A., Kvashnin Y.O., Hellsvik J., Delin A. (2022). Spin-lattice couplings in two-dimensional CrI_3_ from first-principles computations. Phys. Rev. B.

[69] Dupont M., Kvashnin Y.O., Shiranzaei M., Fransson J., Laflorencie N., Kantian A. (2021). Monolayer CrCl_3_ as an Ideal Test Bed for the Universality Classes of 2D Magnetism. Phys. Rev. Lett..

[70] Moriya T. (1960). New mechanism of anisotropic superexchange interaction. Phys. Rev. Lett..

[71] Moriya T. (1960). Anisotropic superexchange interaction and weak ferromagnetism. Phys. Rev..

[72] The DMI parameters (in meV) for the AFE phase are: d⊥1=0.0025, d∥1=0.0066, dz1=0.0323; d⊥2=0.0012, d∥2=0.0054, dz2=0.0162; and d⊥3=0.0010, d∥3=0.0045, dz3=0.0048. For the FE phase, the DMI parameters are: d⊥=0.0070, d∥=0.0070, and dz=0.0203. The orientations of the DMI vectors are shown in Fig. S1. Considering small magnitudes of DMI, we perform rigorous parameter-space evaluations which represents a physically meaningful approach to error estimation in DFT-derived magnetic interactions, where the FE phase is chosen as the example. We find that d⊥=0.0035,d∥=0.0026,dz=0.0272forUeff=1eV;d⊥=0.0050,d∥=0.0045,dz=0.0232forUeff=2eV;d⊥=0.0097,d∥=0.0108,dz=0.0182forUeff=4eV.These results are consistent with Ueff=3eV. The magnitudes of these DMI parameters remain significantly smaller compared to the Heisenberg exchange couplings, implying that variations within this range do not materially affect the outcomes of atomistic spin-model simulations. We then fixed Ueff=3eV and examined the influence of the LDA+Umethods. We obtained d⊥=0.0068,d∥=0.0145,dz=0.0235, which are also consistent with those obtained using the GGA+U and considerably smaller than the corresponding Heisenberg exchange couplings.

[73] Wang X.S., Yuan H.Y., Wang X.R. (2018). A theory on skyrmion size. Commun. Phys..

[74] Gao N., Je S.-G., Im M.-Y., Choi J.W., Yang M., Li Q., Wang T.Y., Lee S., Han H.-S., Lee K.-S. (2019). Creation and annihilation of topological meron pairs in in-plane magnetized films. Nat. Commun..

[75] Kuhn L.T. (2016). In situ transmission electron microscopy for magnetic nanostructures. Adv. Nat. Sci. Nanosci. Nanotechnol..

[76] Tang J., Kong L., Wang W., Du H., Tian M. (2019). Lorentz transmission electron microscopy for magnetic skyrmions imaging. Chinese Phys. B.

[77] Möller M., Gaida J.H., Schäfer S., Schäfer S., Ropers C. (2020). Few-nm tracking of current-driven magnetic vortex orbits using ultrafast Lorentz microscopy. Commun. Phys..

[78] Iacocca E., Liu T.-M., Reid A.H., Fu Z., Ruta S., Granitzka P.W., Jal E., Bonetti S., Gray A.X., Graves C.E. (2019). Spin-current-mediated rapid magnon localization and coalescence after ultrafast optical pumping of ferrimagnetic alloys. Nat. Commun..

[79] Macià F., Kent A.D. (2020). Magnetic droplet solitons. J. Appl. Phys..

[80] Maiden M.D., Bookman L.D., Hoefer M.A. (2014). Attraction, merger, reflection, and annihilation in magnetic droplet soliton scattering. Phys. Rev. B.

[81] Cenker J., Huang B., Suri N., Thijssen P., Miller A., Song T., Taniguchi T., Watanabe K., McGuire M.A., Xiao D., Xu X. (2021). Direct observation of two-dimensional magnons in atomically thin CrI_3_. Nat. Phys..

[82] Bartram F.M., Li M., Liu L., Xu Z., Wang Y., Che M., Li H., Wu Y., Xu Y., Zhang J. (2023). Real-time observation of magnetization and magnon dynamics in a two-dimensional topological antiferromagnet MnBi_2_Te_4_. Sci. Bull..

[83] Allan D.B., Caswell T., Keim N.C., van der Wel C.M., Verweij R.W. (2023).

[84] Bera S., Mandal S.S. (2019). Theory of the skyrmion, meron, antiskyrmion, and antimeron in chiral magnets. Phys. Rev. Res..

[85] Rössler U.K., Bogdanov A.N., Pfleiderer C. (2006). Spontaneous skyrmion ground states in magnetic metals. Nature.

[86] Lu X., Zhu L., Yang L. (2022). Multi-meron interactions and statistics in two-dimensional materials. J. Phys. Condens. Matter.

[87] Jiang L., Tsai H.-L. (2005). Improved two-temperature model and its application in ultrashort laser heating of metal films. J. Heat Trans..

[88] Kresse G., Hafner J. (1993). Ab initio molecular dynamics for liquid metals. Phys. Rev. B.

[89] Kresse G., Furthmüller J. (1996). Efficient iterative schemes for ab initio total-energy calculations using a plane-wave basis set. Phys. Rev. B.

[90] Kresse G., Furthmüller J. (1996). Efficiency of ab initio total energy calculations for metals and semiconductors using a plane-wave basis set. Comput. Mater. Sci..

[91] Blöchl P.E. (1994). Projector augmented-wave method. Phys. Rev. B.

[92] Wang Y., Perdew J.P. (1991). Correlation hole of the spin-polarized electron gas with exact small-wave-vector and high-density scaling. Phys. Rev. B.

[93] Kresse G., Joubert D. (1999). From ultrasoft pseudopotentials to the projector augmented-wave method. Phys. Rev. B.

[94] Smidstrup S., Markussen T., Vancraeyveld P., Wellendorff J., Schneider J., Gunst T., Verstichel B., Stradi D., Khomyakov P.A., Vej-Hansen U.G. (2020). QuantumATK: an integrated platform of electronic and atomic-scale modelling tools. J. Phys. Condens. Matter.

[95] Evans R.F.L., Fan W.J., Chureemart P., Ostler T.A., Ellis M.O.A., Chantrell R.W. (2014). Atomistic spin model simulations of magnetic nanomaterials. J. Phys. Condens. Matter.

[96] Padmanabhan P., Buessen F.L., Tutchton R., Kwock K.W.C., Gilinsky S., Lee M.C., McGuire M.A., Singamaneni S.R., Yarotski D.A., Paramekanti A. (2022). Coherent helicity-dependent spin-phonon oscillations in the ferromagnetic van der Waals crystal CrI_3_. Nat. Commun..

