# Supplementary material for: Controllable properties and versatile dynamics of meron topological magnetism in van der Waals multiferroic CuCrP2S6
Source: iScience. 2025 Aug 5;28(9):113291. doi: 10.1016/j.isci.2025.113291 (PMC12391263; doi:10.1016/j.isci.2025.113291)
Supplement: Document S1. Figures S1–S12 and Table S1 [file mmc1.pdf]

iScience, Volume *28*

Supplemental information

Controllable properties and versatile dynamics of meron topological magnetism in van der Waals multiferroic $CuCrP_2S_6$

Qirui Cui, Yuqing Ge, Xiaocheng Bai, Yasmine Sassa, and Anna Delin

Supplemental Information for "Controllable properties and versatile dynamics of meron topological magnetism in van der Waals multiferroic $CuCrP_2S_6$"

Qirui Cui[1,2*†], Yuqing Ge[1†], Xiaocheng Bai[3], Yasmine Sassa[1], Anna Delin[1,2,4*]

[1]Department of Applied Physics, School of Engineering Sciences, KTH Royal Institute of Technology, AlbaNova University Center, SE-10691 Stockholm, Sweden.
[2]Swedish e-Science Research Center, KTH Royal Institute of Technology, SE-10044 Stockholm, Sweden.
[3]School of Science, Xi'an University of Posts and Telecommunications, Xi'an 710121, China.
[4]Wallenberg Initiative Materials Science for Sustainability (WISE), KTH Royal Institute of Technology, SE-10044 Stockholm, Sweden.

*Corresponding author(s). E-mail(s): qiruic@kth.se; annadel@kth.se;
†These authors contributed equally to this work.

Content:

Supplemental Table S1

Supplemental Figures S1 to S12

Supplemental Videos 1 to 5

Table S1 DFT-resolved Heisenberg exchange couplings (in meV) as functions of U_{eff}.

	AFE			FE
U_{eff} (eV)	J_1	J_2	J_3	J_1
1	1.75	1.58	1.30	2.05
2	1.68	1.49	1.21	1.92
3	1.62	1.42	1.12	1.82
4	1.57	1.33	1.03	1.72

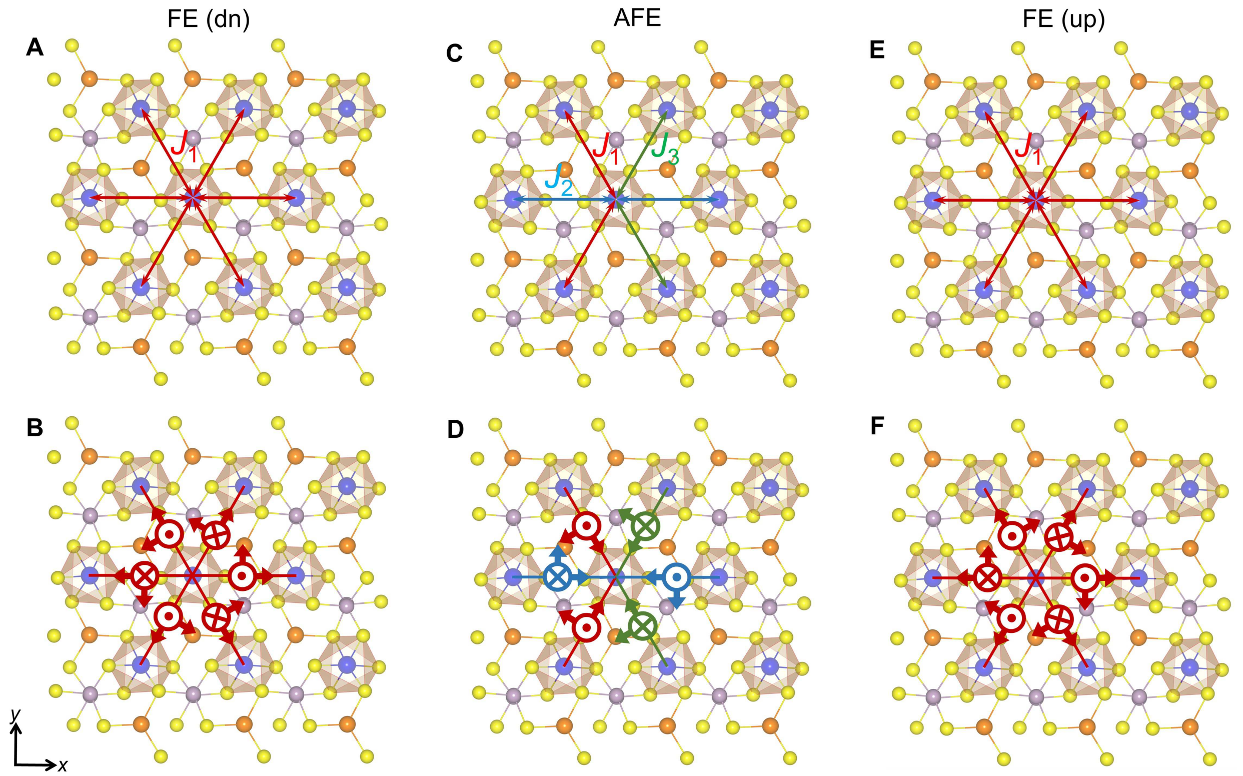

Figure S1. The spin-spin interactions in $CuCrP_2S_6$ (CCPS). The real-space distribution of Heisenberg exchange couplings and Dzyaloshinskii-Moriya interactions is shown for (A, B) the ferroelectric (FE) phase with down polarization, (C, D) the antiferroelectric (AFE) phase, and (E, F) the FE phase with up polarization. In the AFE phase, the Cr atoms are slightly displaced from the centers of sulfur octahedra, where the six nearest neighbors can be grouped into three types with Cr-Cr distances of 5.923 Å, 5.935 Å, and 5.949 Å, respectively.

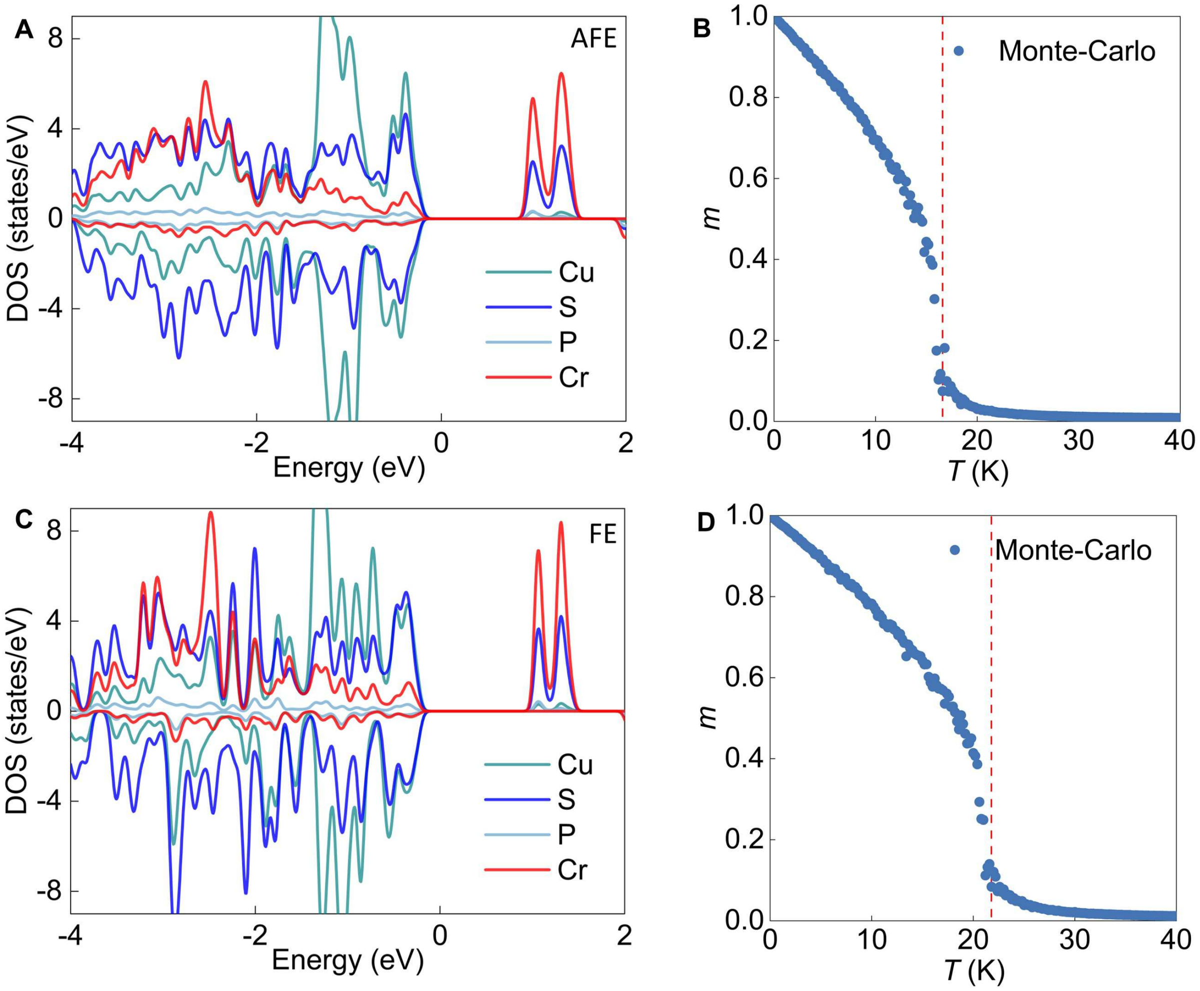

Figure S2. The density of states and temperature-dependent magnetization. Projected density of states for the (A) AFE phase and (B) FE phase. The magnetism of CCPS mainly originates from the spin-polarized 3*d* states of Cr. Compared to the AFE phase, the electronic states in the FE phase become more localized. Temperature-dependent magnetization obtained from Monte Carlo simulations are shown for the (C) AFE phase and (D) FE phase.

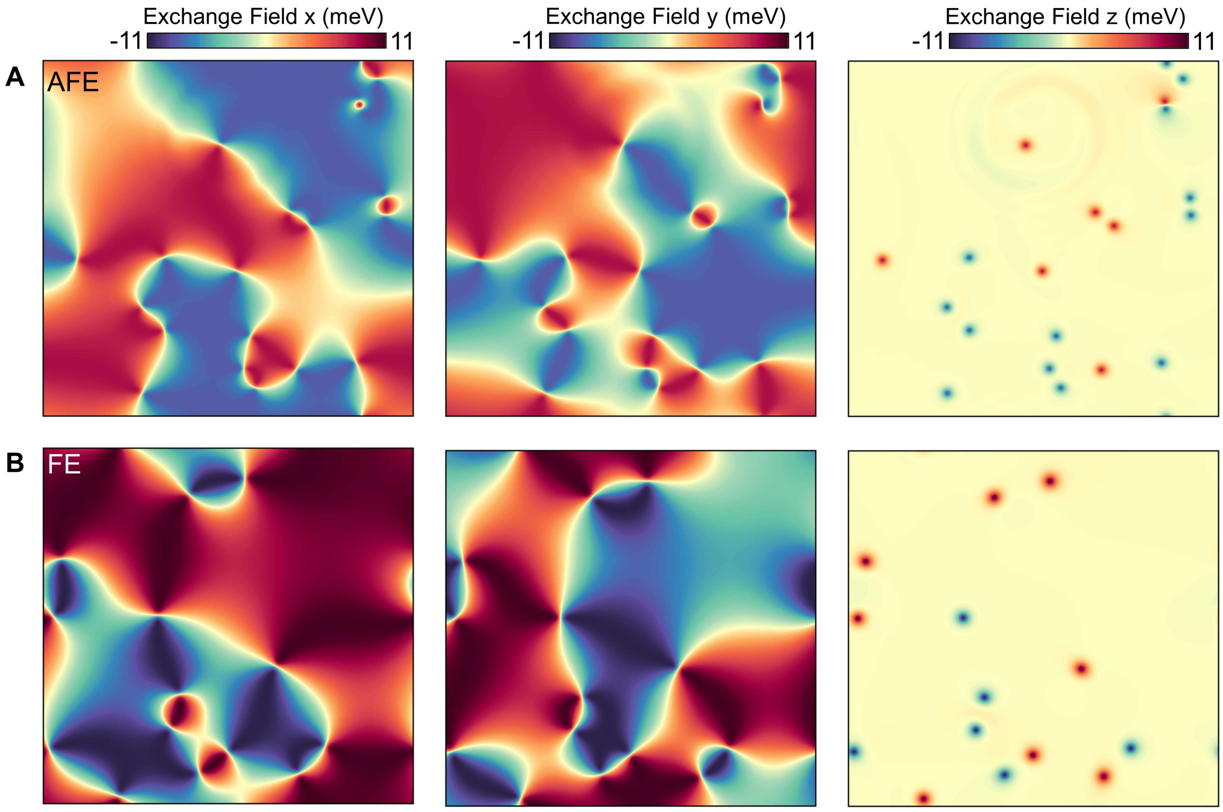

Figure S3. The Heisenberg exchange couplings-driven the formation of merons and antimerons. The projection of the exchange field along the x, y, and z directions for the (A) AFE phase and (B) FE phase, corresponding to snapshots of the spin configurations at $t = 2500$ ps and $T = 0$ K.

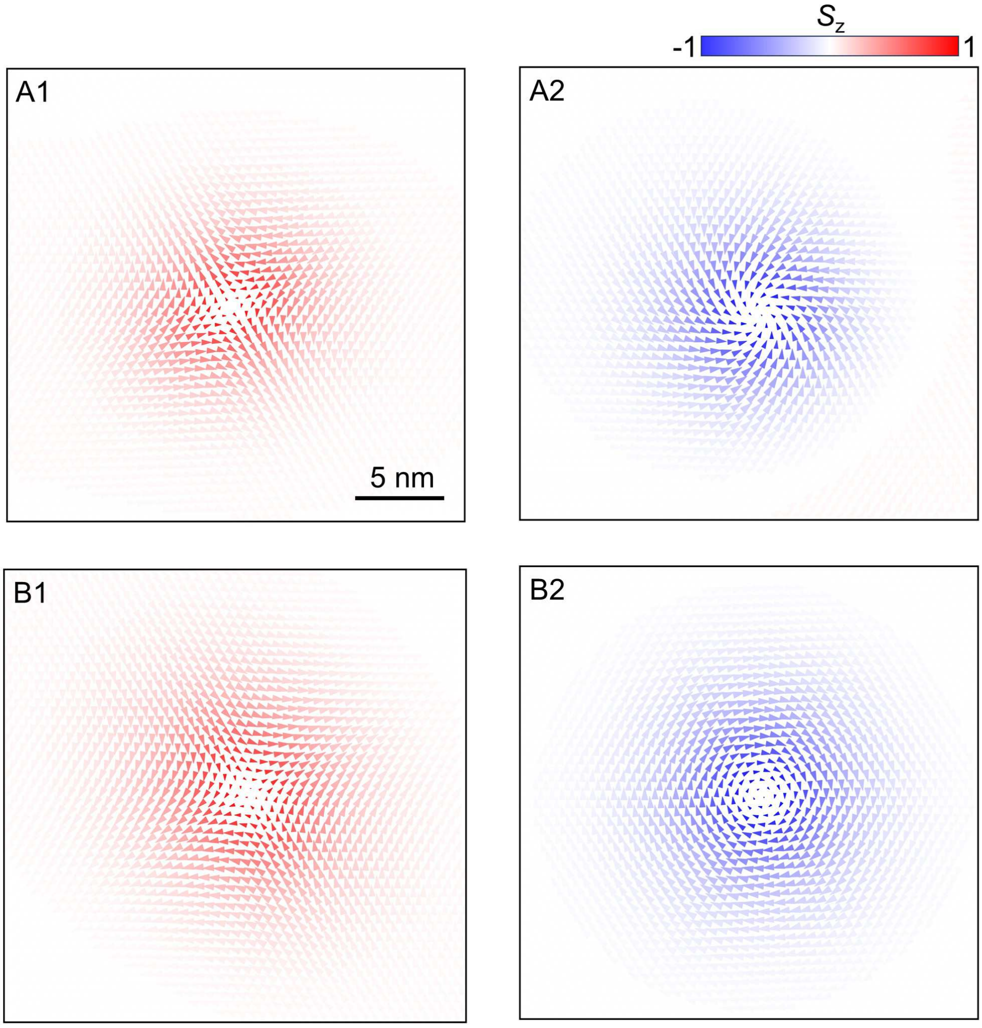

Figure S4. Details of the in-plane spin vectors for merons and antimerons marked by the dashed squares in Fig. 2(B) and 2(E).

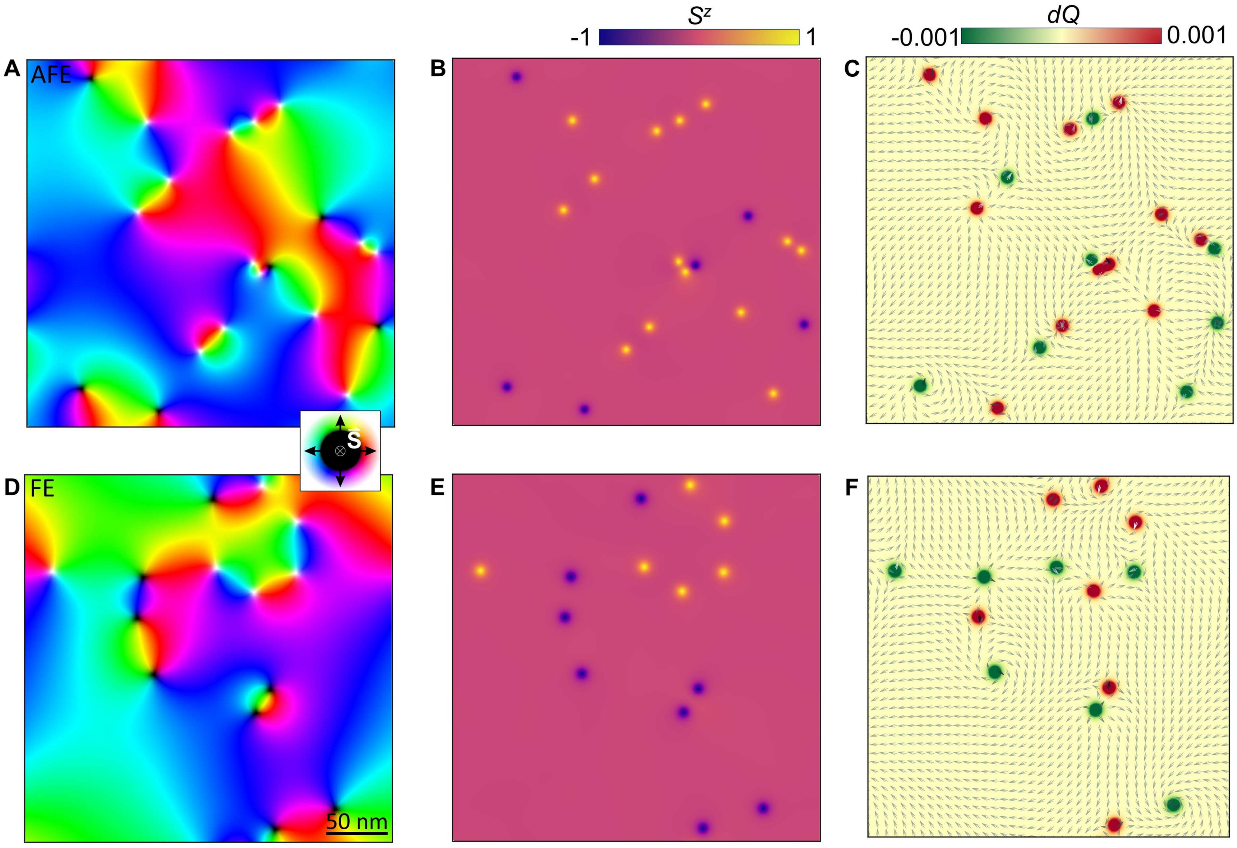

Figure S5. Merons and antimerons in CCPS without accounting for DMIs. (A) In-plane and (B) out-of-plane spin components of the AFE phase at t = 2500 ps and T = 0 K. (C) The corresponding topological charge density. (D)-(F) correspond to (A)-(C), but for the FE phase. Different from the Fig. 2(A)-(F), these spin configurations are resolved without including the Dzyaloshinskii-Moriya interactions in the atomic spin model.

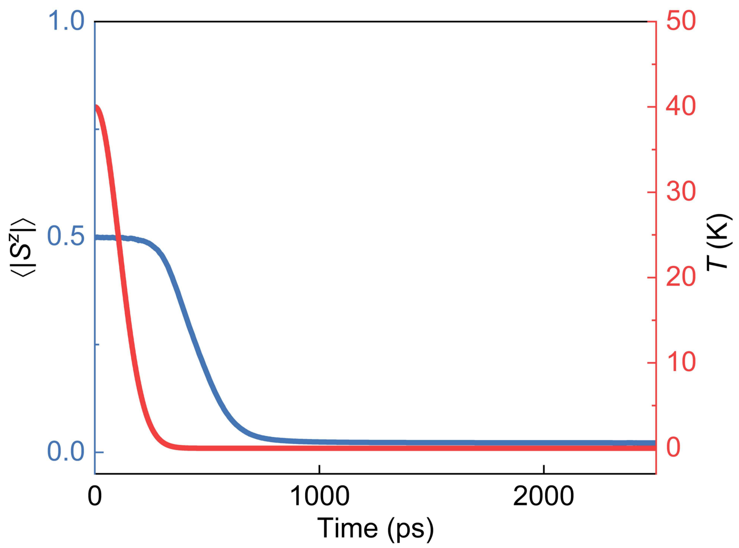

Figure S6. The exchange couplings absence-induced the collapse of out-of-plane spin components. The evolution of temperature and the normalized average of the S^z absolute value, during the zero-field cooling, are presented. The magnitude of the Heisenberg exchange couplings is artificially set to zero, and a random initial state is chosen for the simulations. The S^z component dramatically diminishes as the temperature approaches zero.

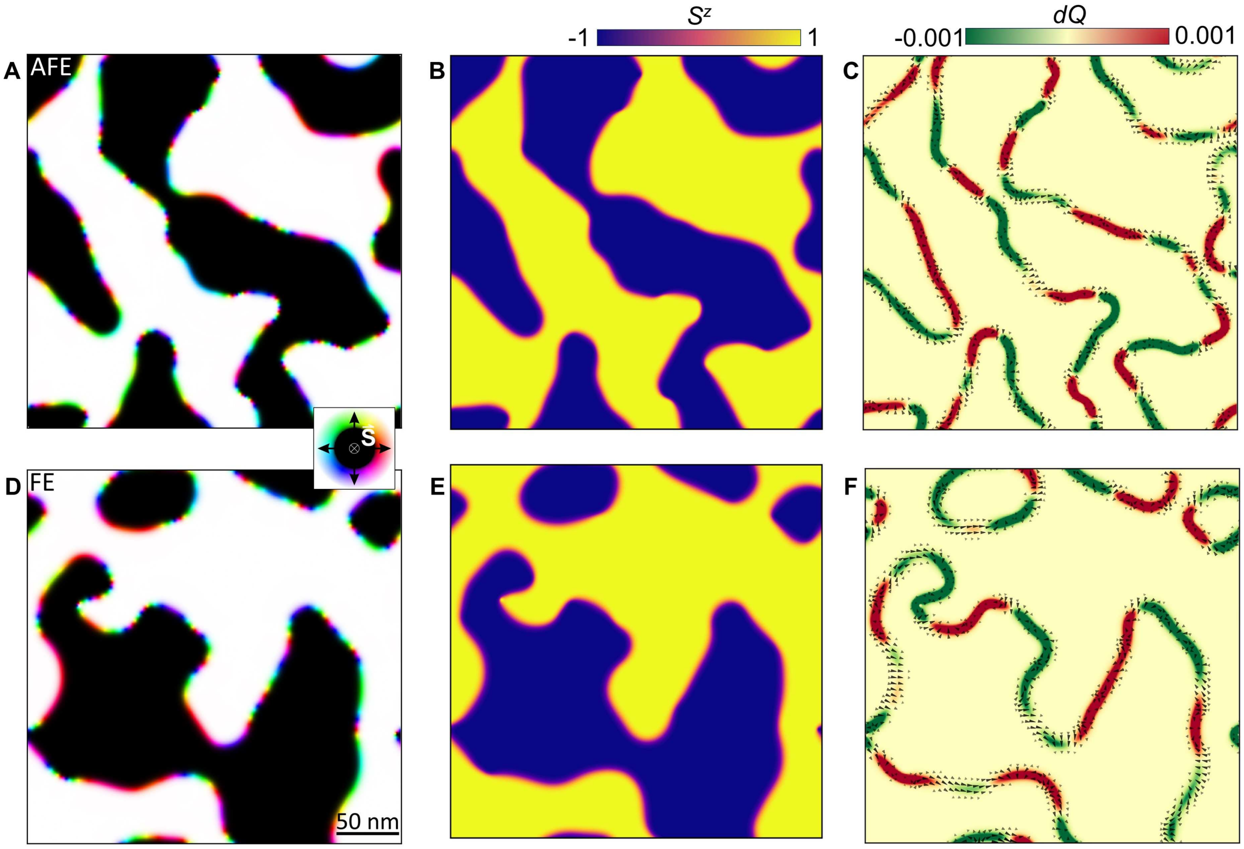

Figure S7. Multiple domains in CCPS with out-of-plane magnetization. (A) In-plane and (B) out-of-plane spin components of the AFE phase at $t = 2500$ ps and $T = 0$ K. (C) The corresponding topological charge density. (D)–(F) correspond to (A)–(C), but for the FE phase. Unlike Fig. 2(A)–(F), the magnetic anisotropy is artificially set to $K = 0.053$ meV for the AFE phase and $K = 0.045$ meV for the FE phase, which favors out-of-plane magnetization. Instead of merons and antimerons, only multiple domains separated by hybridized chiral domain walls are observed.

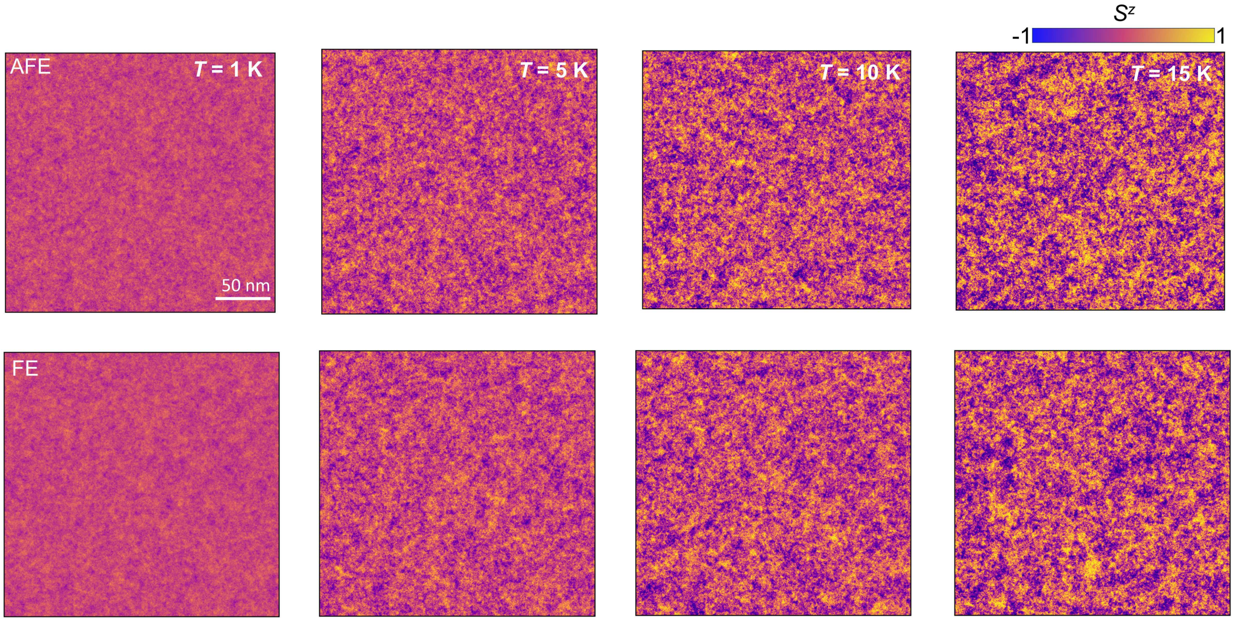

Figure S8. The impact of temperature on the uniform magnetization. Snapshots of spin configurations obtained from time-series simulations under a series of constant temperatures. The initial magnetization is set along the [100] direction. As the temperature increases, the color map becomes more and more blurred due to the enhanced thermal fluctuations.

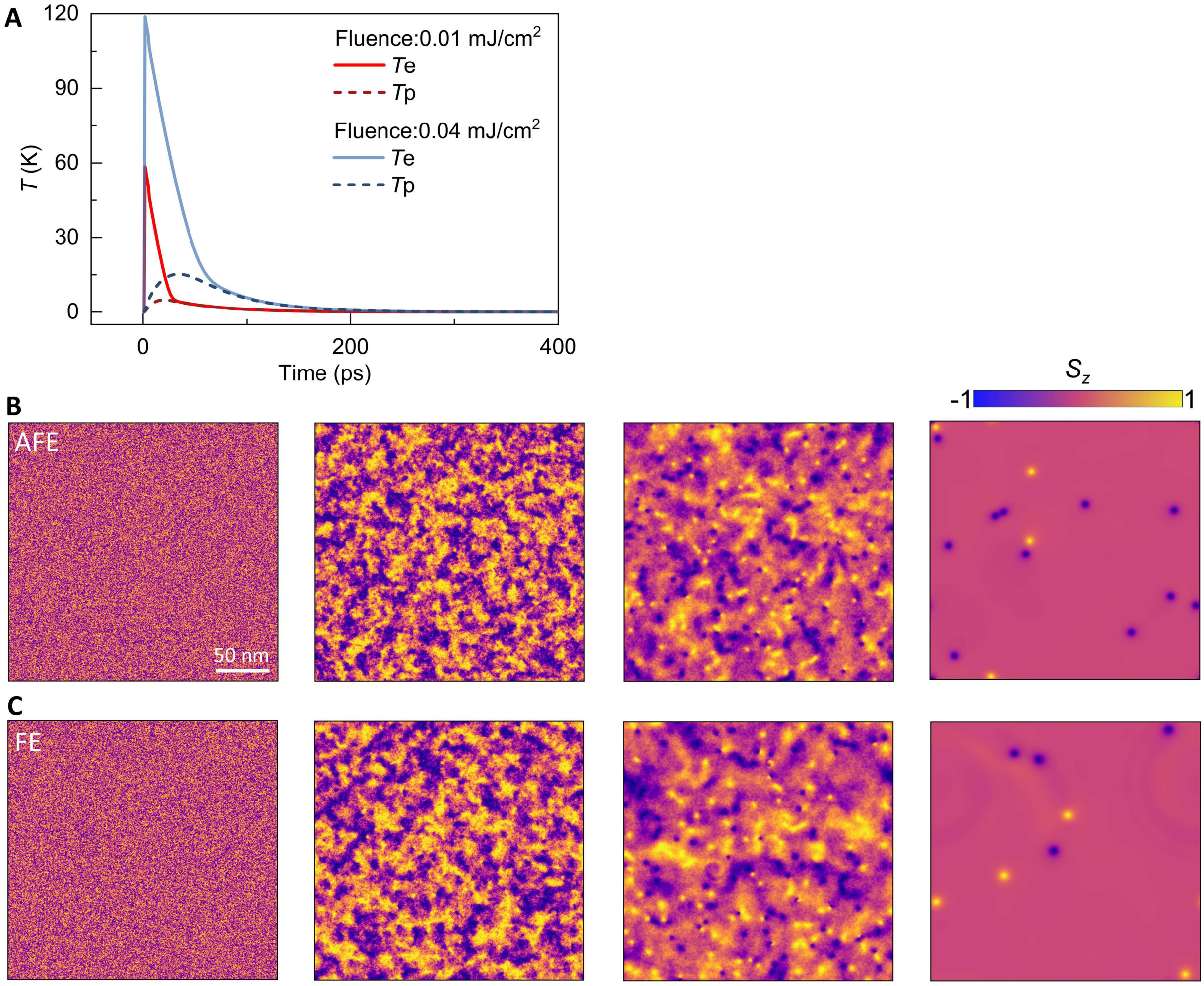

Figure S9. Ultrafast laser pulses-induced merons and antimerons. (A) The electron and phonon temperature response to a 100 fs laser pulse. As the laser fluence is enhanced from the 0.01 mJ/cm^2 to 0.04 mJ/cm^2, more significant thermal fluctuations are induced, resulting in a longer cooling time for the system to reach thermal equilibrium. (B) and (C) display the corresponding spin configurations at different times ($t = 10, 100, 200, 2000$ ps) for the AFE and FE phases, respectively.

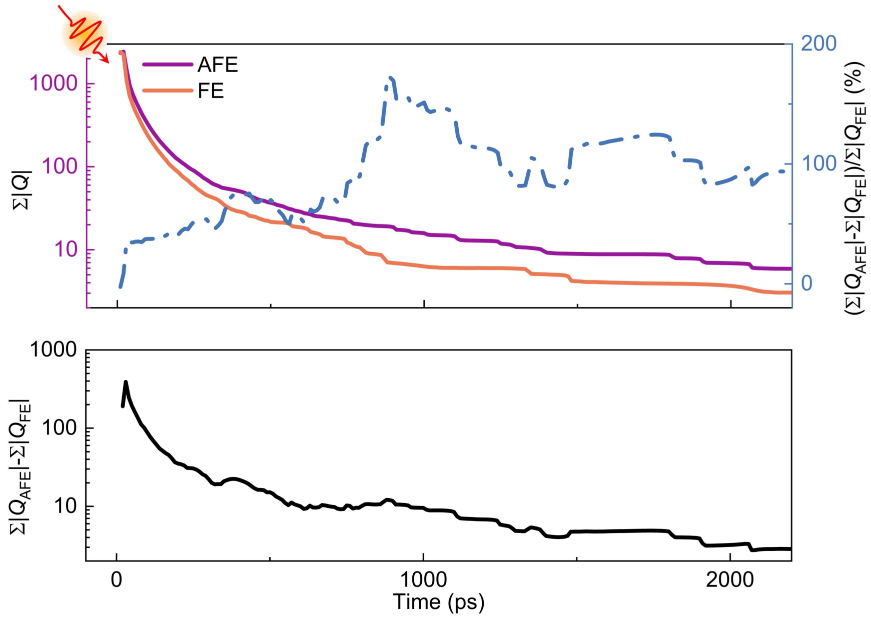

Figure S10. Ultrafast laser pulses-induced meron density difference. After a 100 fs laser pulse with a fluence of 0.01 mJ/cm^2 applied to CCPS with uniform in-plane magnetization, a significant meron number difference between AFE and FE phases is re-induced. The upper panel shows the evolution of the summed absolute values of the topological charge, $\sum |Q|$ (solid lines), and the relative magnitude difference between AFE and FE phases (dashed line) as functions of time. The lower panel highlights the $\sum |Q|$ difference between the AFE and FE phases. Before the laser excitations, both $\sum |Q_{\mathrm{AFE}}| = 0$ and $\sum |Q_{\mathrm{FE}}| = 0$.

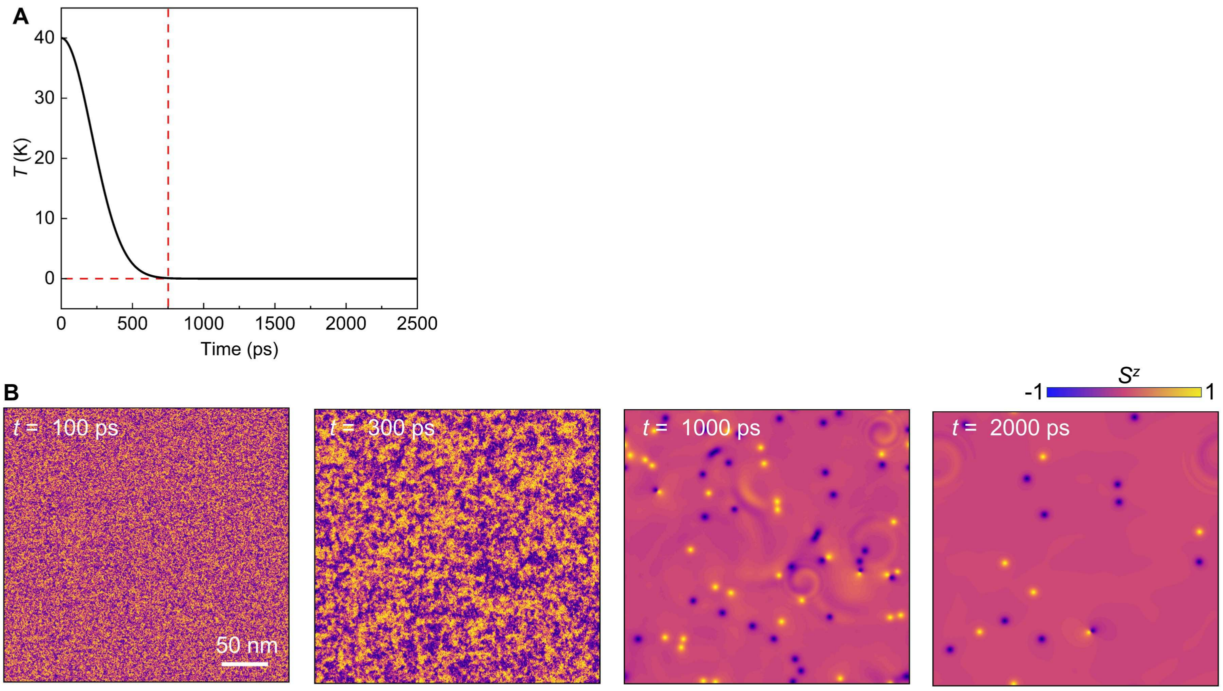

Figure S11. Zero-field cooling simulations of the spin configurations in the AFE CCPS, employing an extended cooling duration of 600 ps. (A) The simulated temperature as a function of time. (B) The time evolution of out-of-plane spin components.

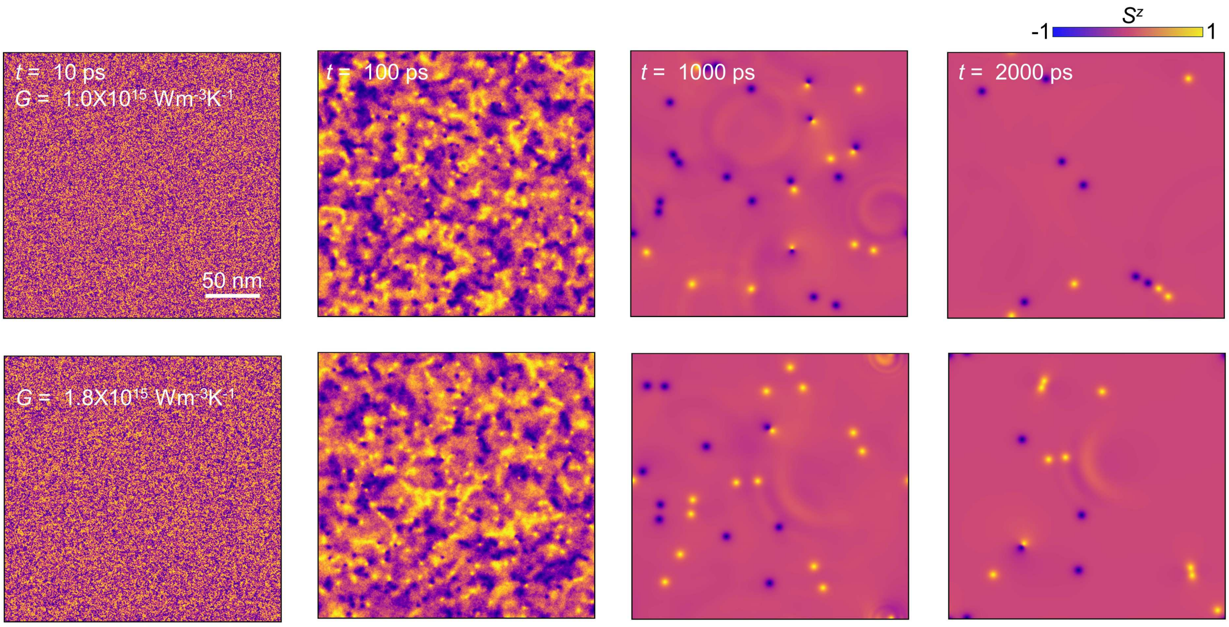

Figure S12. The time-dependent spin configurations for the AFE phase with electron-lattice coupling factor $G = 1.0 \times 10^{15}$ and $G = 1.8 \times 10^{15}$ $\mathrm{Wm^{-3}K^{-1}}$, respectively, in response to the laser. The duration time of the laser pulse is set to 100 fs, and the fluence is set to 0.01 mJ/cm^2. The simulation zone, with periodic boundary conditions, is set to 250×250 nm.
